# Integration of Membrane Proteins into the Outer Membrane of Diderm Bacteria by the BAM Complex

**DOI:** 10.1021/acs.chemrev.5c00764

**Published:** 2026-03-24

**Authors:** Daniel Birtles, Katherine L. Fenn, Jonathan M. Machin, Sheena E. Radford, Neil A. Ranson

**Affiliations:** Astbury Centre for Structural Molecular Biology, School of Molecular and Cellular Biology, Faculty of Biological Sciences, 4468 University of Leeds, Leeds LS2 9JT, U.K.

## Abstract

Assembly of the outer membrane (OM) of diderm bacteria is coordinated by the essential β-barrel assembly machinery (BAM) and is critical for cellular survival and pathogenicity. BAM operates in a membrane environment that is highly rigid and spatiotemporally organized, and functions without ready access to an energy source. In addition, BAM interacts with many other proteins to efficiently fold outer membrane proteins (OMP), assemble complexes in the OM, and maintain cell envelope homeostasis. In recent years, great strides have been made toward understanding the molecular mechanism of BAM-mediated (OMP) folding, with structural biology used to visualize the different stages of the pathways of OMP folding and membrane insertion. The conformational cycling of BAM and its ability to transiently form hybrid barrels with substrate OMPs facilitates their folding. Both these mechanistic features appear to be well conserved and are attractive targets for antimicrobials.

## Graphical abstract

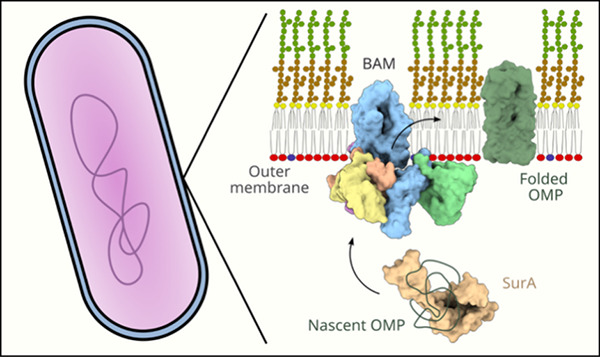

## 1 Introduction

Bacteria are commonly grouped morphogenically based on whether they have a single (monoderm) or double (diderm) membraned cell envelope, historically arising from the use of the gram-stain. (Monoderm bacteria typically stain gram-positive, due to their thick cell wall taking up the stain, while diderm bacteria have a much thinner cell wall and typically stain gram-negative).^1^ Diderm bacterial cell envelopes consist of an inner membrane (IM) and an outer membrane (OM), and an interstitial space (the periplasm) that includes the peptidoglycan cell wall. A healthy cell envelope is essential for proper cell growth, overcoming environmental stresses, and resisting antibiotics and mechanical stress. β-barrel outer membrane proteins (OMPs) form a key part of this barrier, crucial for processes as diverse as OM biogenesis and homeostasis, cell signaling, substrate import and export, cell adhesion, immunity, and mechanical strength.^2^

OMP folding into the OM is catalyzed via the β-barrel assembly machinery (BAM), which in *Escherichia coli* (*E. coli*) is composed of the OMP BamA (consisting of five N-terminal polypeptide transport associated (POTRA) domains, and a 16-stranded β-barrel transmembrane domain) and four lipoproteins: BamB, BamC, BamD and BamE. Given the diverse, critical roles of OMPs and the OM in diderm bacteria, it is unsurprising that BAM is both essential and strongly conserved within diderms. Indeed, mechanistically similar homologues are found across both diderm prokaryotes and in the mitochondria/chloroplasts of eukaryotes.^1,3,4^ Here, we proceed with an overview of diderm diversity alongside BAM conservation and evolution, before focusing in detail on the OM and the foldase activity of BAM in *E. coli*.

### 1.1 Diderm Bacterial Diversity, Yet Omp85 Conservation

The diderm cell envelope architecture is evolutionarily ancient among bacteria.^5^ Current state of the art phylogenetic analysis indicates that the last common bacterial ancestor was a diderm, with an early split forming two clades: Gracilicutes and Terrabacteria. The Gracilicutes clade, which includes *E. coli* and is overwhelmingly more studied, is a monophyletic diderm group. In contrast, the Terrabacteria are a mixture of monoderms and diderms, with monodermicity having independently emerged multiple times. It is thus unsurprising that the cell envelope, especially among the Terrabacteria, has been widely diversified to support specific environmental niches.^1^ A broad array of membrane architectures have been observed, including gross differences in lipid and protein composition,^6^ the width of the periplasm^7^ and/or the peptidoglycan layer,^8^ the porosity of the peptidoglycan layer,^9^ detaching of the OM from the cell wall,^10^ and the presence of an additional outer S-layer^9^ or inner intracytoplasmic membrane.^11^ (Several reviews on cell envelope diversity have recently been published, see refs 1,12 ). While multiple mechanisms for OM biogenesis have been proposed,^6,13,14^ it is unclear how the OM emerged, or how the observed diversity in the cell envelope links to specific cellular function and/or lifestyle.

Among the huge diversity found in OM composition and organization across diderms, two protein families are universally conserved (excluding the diderm species of *Actinobacteria* that possess a mycomembrane): Omp85 (responsible for OMP folding and assembly into the OM) and LptD (responsible for transport of lipopolysaccharide (LPS) into the OM).^1,15^ Indeed, the presence of an Omp85 protein, of which BamA is a member, is one of the best genetic markers for didermicity, and although the family has since functionally diversified beyond OMP assembly with at least 12 different members, it is likely that the archetype was a BamA-like protein.^16^ Omp85 proteins share common structural elements: a 16-stranded transmembrane β-barrel accompanied by one to seven soluble POTRA domains.^17^ The utility and versatility of the membrane insertion function of this protein family is emphasized by its strong sequence and structural conservation, not just among diderm bacteria, but also in mitochondria (SAM) and chloroplasts (Toc75/OEP80).^3^ Coupled with the presence of Omp85 family proteins, their substrate β-barrel proteins are also present across all diderms including the strongly conserved Lpt family and OMP peptidoglycan tethers, albeit with different tethering systems for Gracilicutes (OmpA) and Terrabacteria (SlpA/OmpM). Intriguingly, *Actinobacteria* diderms that have lost their ancestral OM and subsequently re-evolved a distinct mycomembrane OM, also contain (distinct) OM β-barrels, highlighting the utility of this protein architecture, although it is unclear how *Actinobacteria* insert their OMPs into the membrane.

In contrast to the Omp85 family, other subunits of BAM are variously conserved, and their occurrence is limited to the Gracilicutes lineage. Of the four lipoproteins in *E.* coli (BamB-E), BamD is the most ancient and is highly conserved among Gracilicutes, while BamB, E and C arose within the proteobacteria (BamC is the most recent, with it only found in β-/γ-proteobacteria).^18^ BAM from most other Gracilicutes also appears to consist of a minimal BamAD,^19^ with varying additional subunits, for example, BamF in the BamC-less α-proteobacteria and RmpM in *N. meningitidis*.^18,20^ Recent work has structurally characterized BAM from multiple *Bacteroidetes* species, revealing a BamAD core, an essential transmembrane component, multiple surface exposed lipoproteins, and in one case an additional periplasmic component.^21,22^ This distinctive architecture may reflect an adaptation of BAM to the high number of surface-exposed lipoproteins in *Bacteroidetes*, facilitating their export and association with their OMP partners. Together, this highlights that while OMP folding appears to share a common mechanism conserved via BamA/BamAD, by altering its subunit composition the BAM complex has been adapted for distinct folding/assembly challenges, facilitating survival. Notably, BamA functions within Terrabacteria in the absence of any additional lipoprotein subunits (although there may be unknown soluble factors), suggesting that they arose in response to emerging challenges in OMP folding. Intriguingly, both mitochondrial and chloroplast BamA homologues contain additional subunits, suggesting an evolutionary functionalization to their highly specific niche.

Despite the exponential increase in available genetic data in recent years that has facilitated phylogenetic analyses in unprecedented detail, many bacterial phyla remain poorly represented. As more genetic data become available, better phylogenetic trees will enable a clearer view of the last universal bacterial ancestor, and thus a better understanding of the ancestral BamA and its OM context, informing on minimal requirements for function, and potentially generating testable hypotheses regarding the biogenesis of the OM and the diversification and complexification of the BAM complex within it.

### 1.2 Making Up the OM: Lipids, Proteins, and Lipoproteins

While there is huge diversity in cell envelope organization across diderms, many of these architectures remain unstudied in any detail. In all cases the crucial BamA-like component is conserved, and the asymmetric LPS-containing OM architecture akin to that of *E. coli* predominates within bacteria (it is likely typical in at least two-thirds of known phyla^1,15^ ). Hence we focus here on the OM and BAM within *E. coli*. The *E. coli* cell envelope consists of the inner membrane (IM), a periplasmic space 24 ± 5 nm^23,24^ wide containing a thin peptidoglycan layer (3–7 nm wide in *E. coli*) and the OM. The IM is a canonical phospholipid membrane bilayer and is energized by the proton motive force (PMF). BAM meanwhile functions within the OM, which is a protein rich, asymmetric lipid bilayer, with a lipid-to-protein ratio (LPR) as low as ∼ 8:1 ^25–27^ (by contrast, the LPR for the IM is ∼ 32:1 ^26^) (Figure 1). While the inner leaflet of the OM is composed of the lipids phosphatidylethanolamine (PE), phosphatidylglycerol (PG) and cardiolipin in an approximate 80:15:5 ratio,^28^ the outer leaflet lipid is comprised exclusively of LPS.^29,30^ LPS is a complex macromolecule, composed of 4–7 acyl chains of various lengths (typically 12–14 carbons) joined by a diglucosamine (the Lipid A moiety),^31^ and an extracellular glycan chain consisting of a well-conserved 6–7 sugar moiety close to the cell (the core sugars) and a highly diverse repeating unit extending away from the membrane (the O-antigen).^32,33^ Under normal conditions, multiple phosphorylation sites lend LPS a strong negative charge.^34^

**Figure 1:**
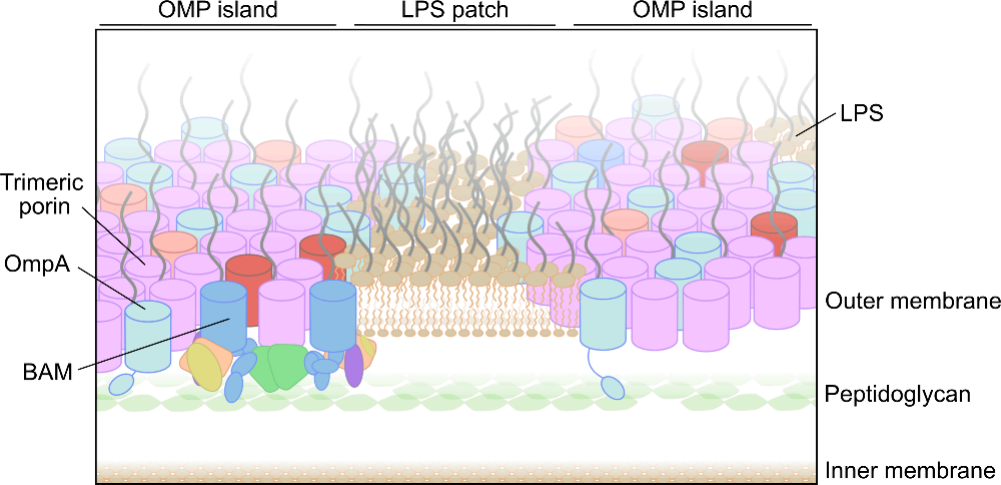
Architecture of the *E. coli* outer membrane (OM). The OM forms the cell surface, which, together with the inner membrane and the peptidoglycan cell wall, comprise the cell envelope. The OM consists of a (partially) phase separated mix of proteins and lipids, within which BAM functions, likely in OMP clusters. Protein arrays are dominated by the OmpF/C trimers which assemble into a hexameric lattice (pink), forming the base of the OMP islands (other OMPs shown in red), while extracellularly extending lipopolysaccharide (LPS) limits access of molecules to the cell. Also shown is OmpA which cross-links to the peptidoglycan layer (cyan). The BAM complex contains BamA (blue), and the lipoproteins BamB (green), BamC (yellow), BamD (orange) and BamE (dark pink).

Membrane asymmetry in the OM is strictly controlled: the Lpt pathway inserts LPS exclusively into the outer leaflet,^35,36^ and mislocalized phospholipids are returned to the inner membrane via the Mla machinery,^37,38^ or degraded via OmpLA.^39^ The proper localization of lipids is essential for maintaining the barrier function of the OM, and phospholipid mislocalization in the outer leaflet sensitizes the cell to otherwise impermeable antibiotics.^40^ Both the Lpt and Mla pathways require OMPs (LptD and OmpC, respectively),^35,38^ which in turn require BAM for their own assembly. The exact mechanisms of phospholipid transport to the OM inner leaflet remain controversial, with the Mla pathway,^41^ AsmA protein family,^42,43^ LetAB^44,45^ and PqiB^46^ all being proposed. *In vitro* studies of OMP folding indicate that folding is kinetically blocked by native lipid headgroups,^47,48^ suggesting that OMPs and the cell envelope membranes have coevolved to ensure folding can only occur via BAM.

Nearly all OMPs are β-barrels.^49^ *E. coli* synthesizes a diverse set of OMPs with monomers ranging from 8 to 26 strands^28^ (up to 36 strands in other species^50^) and oligomers forming barrels of up to 60 strands, often incorporating additional periplasmic or extracellular domains.^51^ Only two of more than 60 OMPs are strictly essential in *E. coli*: LptD and BamA, both of which rely on BAM for folding and membrane insertion^52^ (although loss of others can sensitize the bacterium, e.g. OmpA^53^), highlighting BamA’s role at the center of OM biogenesis. OMPs share some common features: an even number of antiparallel transmembrane β-strands linked by typically short (2–5 residues) intracellular turns and typically long (11–25 residues) extracellular loops and a girdle of aromatic residues at the top and bottom of the transmembrane β-barrel that helps anchor them in the membrane.^54^ A conserved C-terminal sequence motif known as the β-signal is essential for efficient folding,^55,56^ acting as the nucleating strand of a folding OMP in its interaction with BAM (see Section 2.5).^57^ In addition, OMPs have conserved extracellular positive charge adjacent to the membrane (the ‘positive-outside’ rule) that is thought to help support efficient folding.^58,59^

Despite these common elements, substrate diversity in OMP sequences and sizes presents significant challenges to BAM, including protein copy number which varies over 5 orders of magnitude (1–10^5^).^60^ About two-thirds of a cell’s OMPs consist of OmpA and the trimeric porins OmpF/OmpC, which are thought to be present at > 100,000 copies per cell, while there are only perhaps 3–4000 copies of BamA, indicating a folding time of ∼ 10 s (assuming doubling time of 30 min).^60–62^ In contrast, LptD, among the largest and most complicated of the OMP substrates, takes about 20 min to assemble *in vivo*.^63^ Indeed, given the huge diversity in expression levels, it is likely that BAM has evolved to optimize for sufficient flux of high-copy OMPs.

A few OMPs, all oligomeric secretins, do not require BamA to assemble into the OM (Wza, GspD and CsgG in *E. coli*).^64,65^ Intriguingly, unlike BAM-dependent OMPs, PulD (a *Klebsiella oxytoca* GspD homologue) folding *in vitro* is accelerated by native compositions of OM phospholipids, suggesting that its sequence has evolved to enable direct insertion into the OM.^65,66^ This is likely advantageous *in vivo* to facilitate the oligomeric assembly of many subunits (up to 15) in a single pore, which would be challenging if folding were to require BAM. Unlike other OMPs, both Wza and GspD contain regions of transmembrane α-helix,^51,67^ a structural diversity likely afforded by their BAM independent folding. In addition to the oligomeric secretins, there is some evidence that other OMPs (notably the fimbriae ushers) can fold via BAM-independent mechanisms, for example by exploiting the Omp85 protein TamA.^68–70^ However, whether this occurs *in vivo* and, if at all, under what conditions and for what OMPs, remains unclear.

The last major component of the OM are lipoproteins. Despite their relatively recent evolutionary emergence (they are only found in Gracilicutes), they play a crucial role in all OM biogenesis pathways,^71,72^ including OMP assembly (BamB/C/D/E),^73^ LPS insertion (LptE/M),^74,75^ asymmetry maintenance (MlaA),^38^ lipoprotein insertion (LolB),^72^ and stress response (RcsF).^76^ Although some lipoproteins have defined roles, many have no known function or deletion phenotype, indicating niche, as-yet undiscovered roles.^72^ Some lipoproteins are known to be involved in the folding of specific OMPs, with LptM implicated in LptD assembly,^63,75^ while others are thought to help rescue stalled BAM-substrate complexes, for example YcaL.^77^ There is some evidence that certain lipoproteins (e.g., RcsF) can localize to the external face of the OM in *E. coli*,^78^ but there is no clearly defined export mechanism (in contrast to the dedicated machinery that exists in some other bacterial groups, e.g. *Borrelia*).^79^

### 1.3 OM Organization

Exploiting advances in atomic force microscopy (AFM) and super-resolution microscopy, the OM has been revealed as a highly organized, phase-separated structure (Figure 1).^27,80^ OMPs partition into OMP islands, with the trimeric porins OmpF/OmpC forming symmetric arrays, which can be > 500nm across,^81^ while LPS forms its own enriched patches, typically ∼ 55 nm in diameter.^80^ OMP islands are depleted but not devoid of lipid, and LPS is likely important to interface nonspecifically between diverse OMPs.^82,83^ Indeed, LPS is known to have a unique binding fingerprint to a range of OMPs,^84^ and it has been shown to be important for the oligomerization^85^ and function of specific OMPs.^83,86^ LPS has also been suggested to interact with nascent, folding OMPs to help anchor them in the membrane.^87^ OmpA has recently been shown to be critical for proper OMP island formation and thus maintenance of the OM’s barrier function.^53^ Despite these findings, much remains unclear about the nature of LPS-OMP interactions, the relative distribution of OMPs and lipid between the two phases, and the extent of higher levels of organization within the OMP islands, as well as how BAM functions to maintain this organization.

At least in part driven by membrane phase separation, OMP diffusion is typically very slow (∼0.006–0.06 μm^2^/s,^88^ c.f. bacterial elongation rate of ∼ 0.006 μm/s),^89^ however lipid molecules in opposing leaflets of the OM have distinct diffusion properties. LPS in the outer leaflet is essentially immobile, due to its large size and the many arrayed OMPs, alongside cation mediated noncovalent cross-linking between LPS molecules.^90–92^ In contrast, diffusion of lipoproteins in the inner leaflet is more rapid^89,93^ and likely important to allow the formation of OMP-lipoprotein complexes such as the assembly of the BAM complex (as well as other OMP-lipoprotein complexes such as LptD-LptE and OmpC-MlaA). How exactly the BAM complex is assembled following BamA membrane integration remains an open question.

On a cellular scale, OMPs are spatiotemporally organized, with higher levels of insertion at the midcell compared to the poles,^81,92,94,95^ at least partly mediated by inhibition of BAM by mature peptidoglycan^94^ (peptidoglycan is further discussed in Section 1.4). This insertion pattern pushes older OMPs to the cell poles, facilitating binary partitioning upon cell division and thus enabling rapid, but passive, OMP partitioning.^81^ In contrast, LPS is inserted throughout the OM and is only weakly accumulated to the cell poles following division, likely reflecting the less urgent requirement for LPS changes during cell growth and division.^92,96^

BAM has also been shown to cluster in the OM, presumably via association to OMP islands,^92,94,95^ and there is some evidence that multiple copies (∼4–20) of BAM may associate, possibly via the lipoprotein BamB, into ‘folding precincts’.^97^ It is currently uncertain how BAM is organized within OMP islands, and thus whether it natively folds OMPs into lipid-rich or protein-rich membrane domains. The severely limited lateral diffusion of components in the OM creates a membrane packing problem around BAM, where freshly inserted OMPs cannot readily diffuse away from BAM. It is uncertain how this problem is resolved, although the ATP driven insertion of LPS and/or the high thermodynamic stability of OMP folding could provide enough energy to drive components to pack more optimally, but further study is required to understand how the OM is organized and assembled.^92^

### 1.4 The Cell Envelope

The OM is linked to the broader cell envelope – the peptidoglycan, IM and periplasmic space – both physically and via shared pathways, processes and functions. While historically the cell wall (the peptidoglycan layer (Figure 1)) was thought to maintain the mechanical properties of the cell envelope,^98^ the cell’s load-bearing structure has recently been redefined as the peptidoglycan, OM and their cross-linking proteins working in concert.^99,100^ These cross-linking proteins – Lpp (covalently linked to peptidoglycan) and OmpA and Pal (noncovalently interacting with the cell wall) – tightly tether the OM to the peptidoglycan layer, together maintaining the strength, shape and size of the cell envelope.^99,101^

Peptidoglycan is a highly dynamic entity constructed of a cross-linked network of peptide-oligosaccharides that is synthesized, modified, and degraded by ∼ 40 distinct enzymes in the periplasm.^102^ Its synthesis and OMP assembly are tightly integrated, with BAM known to both interact with peptidoglycan and to be inhibited by its mature (cross-linked) form,^94,95^ ensuring that most OMPs are inserted into the OM adjacent to nascent peptidoglycan. Tight spatiotemporal control of peptidoglycan remodelling is necessary to allow for proper cell elongation and division, especially given the thinness of the peptidoglycan layer in *E. coli* (3–7 nm).^103^ This regulation is achieved through both modulatory and localizing protein–protein interactions (predominantly in the elongasome and divisome complexes).^102,104,105^ Intriguingly, the OM lipoproteins LpoA and LpoB are required for the activity of synthase enzymes PBP1A and PBP1B at the IM, suggesting a pathway for changes at the OM to modulate peptidoglycan synthesis, although how this occurs is unknown.^106,107^

The IM sits, on average, 14–16 nm below the peptidoglycan layer and is markedly different from the OM in both its composition and organization. Although the IM is the synthesis site for LPS, due to its active transport to the OM, LPS remains at very low abundance in the IM. Rather the composition of the IM more closely resembles the inner leaflet of the OM, consisting of the phospholipids PE, PG and cardiolipin (∼80:15:5),^108^ with anionic lipids enriched toward the cell poles.^108^ There is some evidence lipids are asymmetrically organized, with their distribution varying with cell size and the cell cycle, especially for PE and cardiolipin.^109^ Unlike the OM’s β-barrels, IM transmembrane proteins are comprised of transmembrane α-helices that are folded and inserted into the IM via the SEC translocon or YidC.^110,111^ In contrast to OMPs in the OM, IM proteins can diffuse freely, due to the higher LPR of the IM (∼32:1)^26^ (which is comparable to the LPR of eukaryotic plasma membranes^112,113^ ) and the lack of immobile LPS.^114^ Some proteins have been shown to cluster into arrays for functional purposes (e.g., signaling^115^) but this is not thought to be a general feature for IM proteins as it is for OMPs. Although there is some evidence of lipid raft formation,^116,117^ a widespread phase separation between the protein and lipid components of the IM has also not been observed. Therefore, while both the SEC translocon and BAM machineries each partition and fold proteins into membranes, their different architectures and functional mechanisms reflect the differing physical characteristics of their substrates and respective membranes.

Dedicated machinery exists for the transport of periplasmic, OM and exported components across the inner membrane. Prior to their IM translocation, most periplasmic proteins, and all OMPs, are targeted to the SEC translocon via the signal peptide, a ∼ 20–25 residue sequence at the protein’s N-terminus consisting of a positively charged sequence followed by a hydrophobic region.^118^ While the SEC machinery translocates unfolded polypeptides destined for the periplasm and OM across the IM it is also responsible for folding and insertion of the majority of the IM’s transmembrane proteins.^110,119^ Crucially, the SEC translocon recognizes a substrate’s hydrophobicity, with highly hydrophobic sequences (typically with α-helical propensity) such as a transmembrane helix or a signal peptide able to open the SEC translocon’s lateral gate and partition into the IM.^118^ The transmembrane β-strands of OMPs are (considered as a whole-strand) less hydrophobic than transmembrane α-helices, likely allowing the SEC translocon to distinguish them from proteins destined for the IM, and facilitate their export.^110,120,121^

Following translocation through the SEC translocon, the IM anchored signal peptide is cleaved off OMPs and periplasm-resident proteins by signal peptidase I (at an AXA motif, where X is any residue) releasing them into the periplasm.^122^ For OM lipoproteins, an invariant cysteine in the lipobox motif is S-diacylated before cleavage of the signal peptide immediately N-terminal to the cysteine by signal peptidase II, and an additional acylation then occurs on the cysteine’s exposed amino terminus.^72^ While the majority of IM-translocated proteins, including all OMPs, are targeted to the SEC translocon for their translocation into the periplasm, a small subset of proteins that require folding in the cytoplasm before being transported make use of the alternative TAT machinery.^123^ Intriguingly, the SEC translocon appears to be active and distributed evenly across the IM, suggesting that OMPs are translocated throughout the periplasm, although most are destined to fold at the midcell where BAM is most active.^92,94^ Once exported through the IM, all molecules destined for the OM, including chaperoned unfolded OMPs, must pass through the peptidoglycan layer. Given that Lpt bridges can form and insert LPS evenly across the cell surface,^92,124^ the peptidoglycan layer must be latticed with sizable holes to facilitate rapid Lpt subunit diffusion, bridge formation and subsequent LPS transport. Different experiments have observed pores of 4–12 nm diameter,^125,126^ sufficient for Lpt bridge formation and for the diffusion of a chaperoned unfolded OMP. Immature peptidoglycan at the midcell has fewer cross-links and thus would be expected to create larger pores,^102^ potentially supporting more efficient diffusion of chaperoned unfolded OMP substrates to BAM, possibly providing an additional explanation of why more OMP synthesis occurs at the midcell.

In addition to the Lpt machinery, which forms transient bridges (lifetime ∼ 10s) across the periplasm,^124^ other cell envelope spanning complexes can form either transiently (TonB dependent transporters:TonB),^127^ long-lived (type I/II/III/IV/VI secretion systems)^128^ or permanently (flagella).^129^ Crucially, these cross-periplasm structures allow energized processes to occur at the OM by coupling them to cellular energy sources, either the cytoplasmic hydrolysis of ATP or inner-membrane PMF. Indeed, the PMF across the inner membrane is essential for a range of periplasmic and OM processes, including active substrate transport across the OM^127^ and cell motility.^130^

Unlike these processes, BAM is generally thought to function without access to an energy source to overcome the energetic barriers of OMP folding (although it has been suggested to couple to IM PMF in some instances).^131^ Beyond this, the unique nature of the OM and periplasm presents myriad other challenges to BAM’s efficient activity: partitioning new OMPs into a highly rigid membrane, maintaining protein/lipid phase separated organization, and assembling multimeric complexes. Furthermore, BAM must interface with lipoprotein and periplasmic partners, as well as peptidoglycan, to facilitate efficient, spatiotemporally controlled folding and quality control mechanisms. Given these challenges, it is remarkable that the BAM complex can successfully function over the breadth of its substrate OMPs without inducing substantial membrane defects.

## 2 The BAM Complex and Folding an OMP

Since the essential function of the BAM complex in *E. coli* was first recognized in 2005,^132,133^ the *E. coli* complex has become the archetype for structural, functional, *in vivo* and *in vitro* studies that aim at understanding OMP folding and assembly into the bacterial OM. Studies on *E. coli* BAM have yielded a wealth information about the OMP folding process, including details of how OMPs are delivered to BAM via the chaperone SurA,^134–137^ OMP β-signal engagement,^55–57^ OMP assembly from C- to N-termini via the sequential formation of β-strands,^138–141^ and the release of fully folded OMPs into the membrane through a zipper-like mechanism.^142^ By contrast, there is only a relatively modest amount of structural and biochemical information available on BAM from other species, with one notable exception from the recent determination of the structure of BAM from *Bacteroidetes*.^21,22^ Hence, we here focus on discussing recent discoveries and mechanistic insights into the BAM complex of *E. coli*. Where relevant, brief mention regarding other BAM homologues is included.

At the core of all BAM complexes is BamA, itself a 16-stranded OMP, and the only transmembrane component of the complex. Nascent BamA requires a pre-existing BAM complex to fold into the membrane,^141^ with the newly folded BamA presumably providing a scaffold for its associated lipoproteins (BamB/C/D/E in *E. coli*) to assemble around, but the molecular details of how the complex assembles remain unclear. In addition to its 16-stranded transmembrane β-barrel domain, BamA has five POTRA domains (numbered 1–5 N-to-C terminally), which form a spiral under the barrel (Figure 2). The four BAM lipoproteins are arranged asymmetrically on BamA: BamB interacts at POTRA-2/3, while BamD and BamE interact at POTRA-5 and the BamA barrel’s first, second and third periplasmic turns. In apo BAM, BamD also interacts at the opposite side of POTRA-2, contacting BamB, together forming a periplasmic ring under the BamA barrel (Figure 2). BamC binds to BamD via an ordered N-terminal loop region followed by two loosely interacting helix-grip domains, which may interact with BamA under certain conditions.^73,143^ How these structural features of BAM are finetuned to facilitate efficient OMP folding is next described.

**Figure 2:**
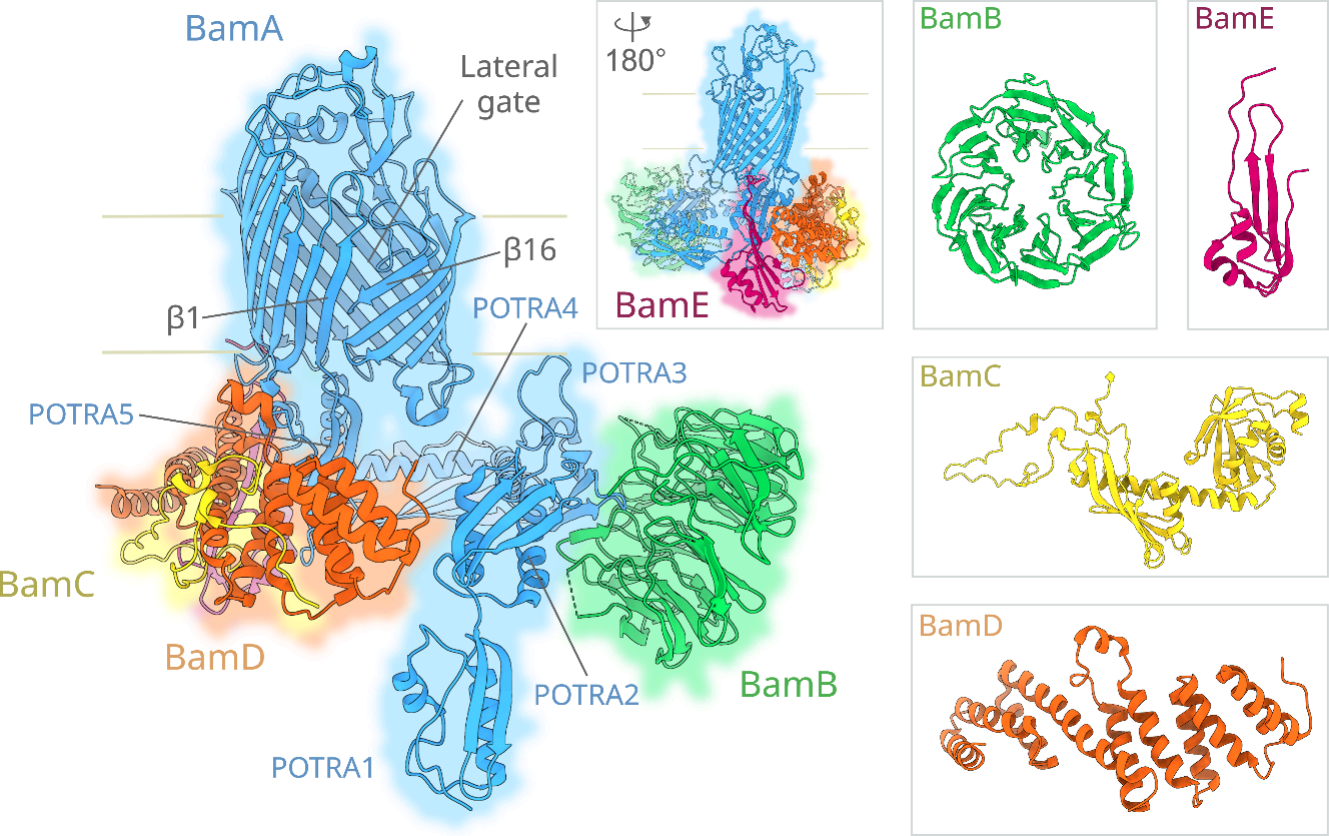
Structure of *E. coli* BAM. *E. coli* BAM is composed of the OMP BamA (blue) and the lipoproteins BamB (green), BamC (yellow), BamD (orange) and BamE (magenta). Composed of a 16-stranded β-barrel and five periplasmic POTRA domains, BamA is an unusual OMP in that its first (β1) and last (β16) β-strands can dynamically open to form a lateral gate facing the membrane (here shown in its ‘Lateral Open’ conformation). The accessory lipoproteins use the periplasmic POTRA domains of BamA as a scaffold to assemble the full complex. The two loosely associated helix-grip domains of BamC are rarely structurally resolved and are not shown in the main complex. (PDB 9CNW).^144^

### 2.1 Investigating BAM Mediated OMP Folding

Elucidating precisely how the BAM complex folds an OMP into the OM is challenging for multiple reasons. BAM has a plethora of substrates, making it difficult to determine which of its features are substrate-specific and which may be common among different clients. Do relatively simple OMPs (e.g 8 stranded monomers) fold on BAM in a similar mechanism to its more complex clients that may have multiple domains, larger barrels and more diverse associated domains on the extracellular or periplasmic sides of the barrel? Such substrate-specific folding features likely influence LptD assembly, which has frequently been used to study OMP folding intermediates due to its slow folding rate.^145–149^ LptD is also an unusual OMP due to its large size (26 β-strands), its requirement for disulphide rearrangement to reach the mature form, and its interactions with additional lipoproteins (LptE and LptM) and hence this substrate, most likely, requires unique adaptations to BAM’s general catalytic mechanism.^63,75,150,151^

Functional studies of BAM have been hindered by additional experimental challenges, such as those associated with replicating the asymmetric OM *in vitro*. Structural studies have generally utilized simpler synthetic membrane mimics such as detergents^136,137,141,143,144,152–158^ and nanodiscs^57,142,144,156,159–161^ (see for a detailed overview of all cryoEM structures solved featuring the full BAM complex to date), while functional studies generally use liposomes created from defined lipids or *E. coli* polar lipid extract.^153,154,159^ As lipids often have crucial roles in membrane protein structure, function and regulation, it is contextually important for such proteins to be investigated in as near native chemical environments as possible. The extremely low LPR (∼8:1) of the OM also poses an interesting conundrum: the ∼ 4-fold decrease in lipids per OMP compared to their IM counterparts might suggest a decreased importance of protein: lipid interactions, yet conversely such interactions may be more significant in the OM due to their limited number.

Folding intermediates are metastable structures that form during the OMP assembly process and require artificial stabilization to be structurally characterized due to their transient and unstable nature. Capturing such intermediates generally involves the engineering of cross-links, either based on prior evidence of an interaction site or to find one, both of which require significant experimental work. Such engineered-cross-links are often accompanied by a deletion of part of the OMP substrate (mainly loops) or truncation of the barrel to prevent the completion of folding.^141,145^ The development and continued improvement of computational tools capable of predicting protein structures and protein–protein interactions is a valuable asset, and one that has already been successfully used to guide the identification of cross-linking sites for the visualization of OMP folding intermediates.^137,162–164^ Although trapping is generally performed in native membrane environments, the effect of reconstitution in detergents (such as glyco-diosgenin (GDN) or n-dodecyl-β-D-maltoside (DDM)), on the overall conformation of the BAM-OMP structure remains unclear and should be considered when interpreting fine structural details. Nonetheless, over the past decade, great strides have been made in elucidating BAM mediated OMP assembly, particularly how the process develops from chaperone delivery all the way through to OMP release.^165,166^ However, several key questions within the mechanism remain unanswered and these are pointed out in the sections below.

### 2.2 BAM Dynamics

BAM is conformationally flexible, and the dynamics of these movements appear to be integral for OMP folding and assembly. Critical dynamic movements occur at the seam of the BamA β-barrel, which can be defined as the site of interaction between the terminal β-strands (β1- β16), as well as in the orientation of the POTRA domains and BamB-E lipoproteins relative to the barrel domain. The idea that BamA might insert OMPs into the membrane via lateral opening was first mentioned more than twenty years ago,^167^ but did not gain any real traction until publication of the first BamA crystal structures (from *Neisseria gonorrheae* and *Haemophilus ducreyi*) in which relatively few contacts were observed between the β-seam, and molecular dynamics (MD) simulations revealed that the barrel could ‘open’ and ‘close’ based upon whether these interactions were maintained.^168^ The two conformations (Lateral Open and Lateral Closed) were experimentally validated by multiple groups, with both structures eventually witnessed in the presence of the complete BAM complex and further details defined, such as the necessity for a C-terminal kink at β16 for lateral gate opening.^73,143,152,169,170^ From a functional perspective, restriction of dynamics at the lateral gate by addition of a disulphide bond was found to prevent efficient OMP assembly *in vitro* and is lethal *in vivo*.^143,168^

In the Lateral Closed conformation of BamA the β-seam is closed via hydrogen bonding between β1-β16, and the lumen of the BamA barrel is exposed to the periplasm (Figure 3a). By contrast, in the Lateral Open conformation the β-seam of BamA is broken and POTRA-5 moves to obstruct the entrance of the BamA barrel lumen to the periplasm (Figure 3b). The movement of POTRA-5 and the availability of the BamA lumen to the periplasm led to the terms ‘Outward Open’ (Lateral Open) and ‘Inward Open’ (Lateral Closed) also being coined to describe its dynamic motions.^73^ These essential dynamics are necessary for the partitioning and release of substrate OMPs.^142,143,168^ However, several questions remain regarding the exact roles of both the Lateral Open and Lateral Closed states, alongside how the significant structural transitions between the two conformations facilitate OMP assembly.

**Figure 3:**
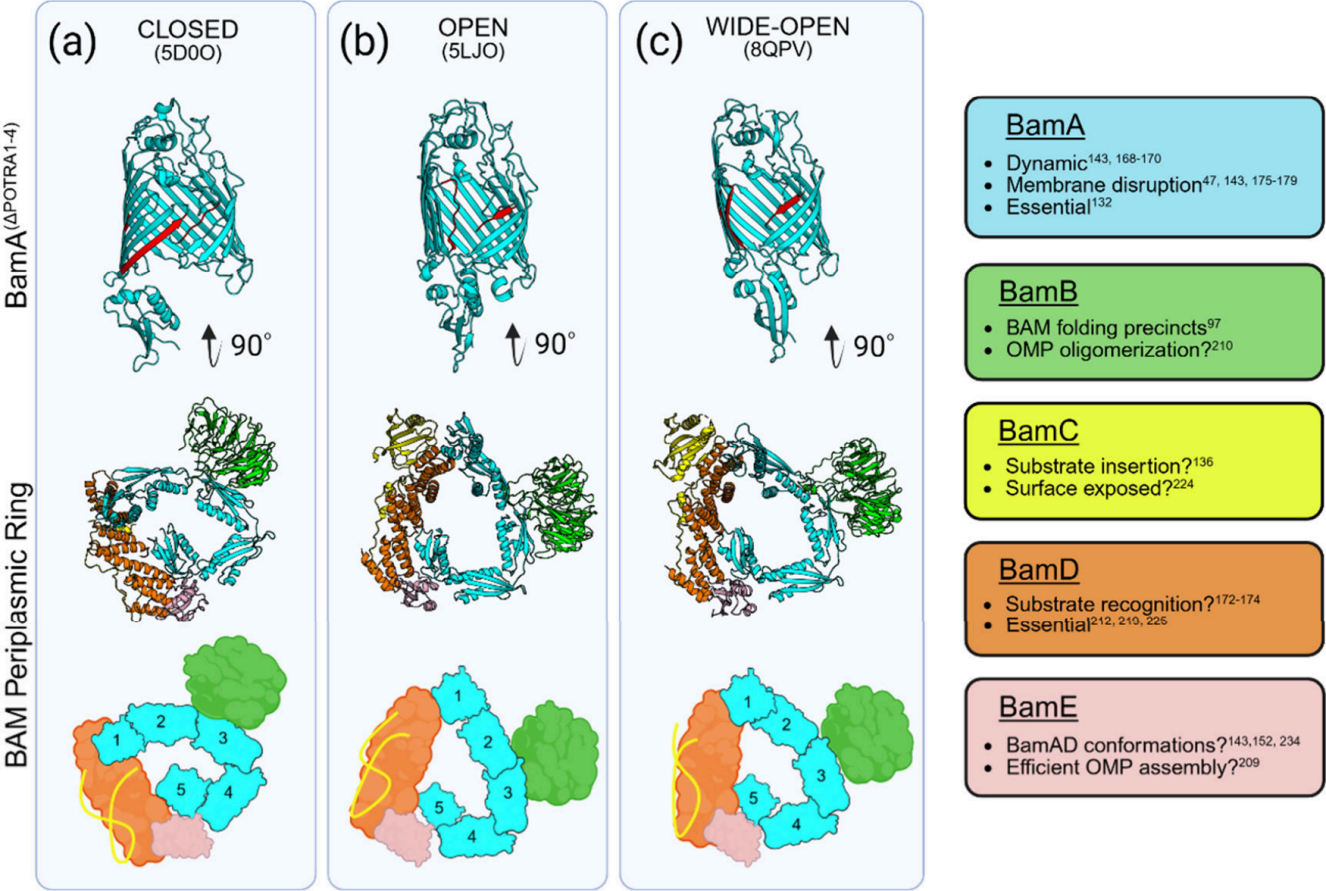
BAM dynamics. (a) In the Lateral Closed conformation of BamA, β1 (Red) and β16 (Magenta) are associated and the lumen of the barrel is accessible. (b) Upon Lateral Opening, β1 and β16 dissociate, POTRA5 occludes the periplasmic entry into the BamA barrel, and the POTRA domains rotate ∼ 30°, rearranging the BAM lipoproteins. (c) In the Wide-Open conformation the distance between β1 and β16 increases still further compared with the Lateral Open state, while only minor changes in the POTRA domains are detected. Only components visible at high resolution are shown in all three states in the bottom schematic for clarity. Roles of the individual BAM proteins are also summarized in the key alongside, with speculated functions highlighted via a question mark. Created in BioRender. https://BioRender.com/395lm3g

The transition from Lateral Closed to Lateral Open involves substantial structural reorganization of every component within the BAM complex. At the β-seam of BamA, β1 rotates ∼ 65° together with β2-β6 as it separates from β16, with the extracellular region of β1 moving ∼ 10–15Å away from the barrel lumen.^73,152^ In addition, the periplasmic end of β1 moves toward the lumen, bringing with it POTRA-5 to occlude the base of the BamA barrel in the Lateral Open conformation. At this point the periplasmic ring has rotated by ∼ 30° around the barrel (Figure 3b), with the POTRA domains also vertically extending away from the membrane.^143^ A hinge between POTRA-2 and POTRA-3 appears to be key for this conformational cycling of the domains that is required for function.^171^ ( – )

Apart from BamC, whose helix-grip domains are dynamic and not modeled in most high resolution cryoEM structures of BAM, the remaining BAM lipoproteins exhibit little movement as the lateral gate opens and closes, other than that driven by their individual interactions with the POTRA domains. Minor rearrangements within BamD are thought to support the conformational dynamics of BamC and the restraining of POTRA-1 and POTRA-2 in the open conformation.^136^ Unfortunately, little is currently understood about the contributions such motions make to BAM function, with even less identified regarding how the role of the lipoproteins are impacted by their own dynamic capacity as well as that of the complex.^172–174^

### 2.3 BAM Membrane Distortion

Alongside dynamics, the ability of BAM to disrupt the local membrane environment is also thought to play an important role in BAM-catalyzed OMP folding. The asymmetric shape of the BamA β-barrel, which is governed by differences in the hydrophobic width of the transmembrane section, has been shown through structural elucidation and MD simulations to thin and distort the membrane local to the β-seam across a range of diverse species.^47,159,168,175–179^ Membrane thinning has also been observed toward the back of the BamA barrel (β8-β9), although to a lesser extent than at the β-seam.^179^ Studies *in vitro* further established the importance of the physical membrane properties in determining the rate of OMP folding. Specifically, the spontaneous insertion rates of OMPs are generally increased in lipid membranes with increased fluidity, decreased thickness and in the presence of membrane defects.^48,180^ Membrane distortion by BAM is thus likely a fundamental component of the molecular mechanism(s) necessary to facilitate OMP folding and insertion.

In addition to the local membrane disruption attributed to BamA in nanodiscs,^159^ a more global destabilization of the lipid bilayer was found in DMPC liposomes containing BAM.^153^ Intriguingly, BamA alone does not mirror this behavior, implicating at least one of the BAM lipoproteins in this effect. While the extent or functional significance of membrane destabilization in vivo is currently unknown, such an effect presumably would facilitate OMP insertion into the bilayer. Thus, structural asymmetry of the BamA barrel combined with the conformational changes undergone by the BAM complex likely work in unison to prepare the local membrane environment for efficient OMP insertion.

### 2.4 SurA and OMP Delivery to BAM

The delivery of OMP substrates to BAM is the first major step in BAM-mediated OMP biogenesis. In the periplasm, chaperones bind to unfolded OMPs, maintaining them in a folding competent state delivering them to BAM for folding and insertion into the OM. There are several periplasmic chaperones that play different roles in OMP and periplasmic protein folding, including SurA, FkpA, Spy and Skp, all of which operate in the absence of ATP.^181,182^

#### 2.4.1 SurA Chaperones OMPs in the Periplasm

The major chaperone responsible for OMP biogenesis is SurA.^134,135^ Its importance in integrating OMPs into the OM is highlighted by the dramatic reduction of all major OMPs in proteomics of ΔSurA strains concurrent with a broad activation of the σ^E^ response (which is invoked when there is an increase in unfolded proteins in the periplasm).^135,137,183^ The subsequent loss of OM integrity in these strains means they have increased susceptibility to large antibiotics such as vancomycin, which is normally only effective against monoderm bacteria.^137,184,185^

SurA must bind the wide variety of OMPs to prevent their aggregation in the periplasm. SurA binds its OMP clients with low (micromolar) affinity,^163,186–191^ but the exact binding specificity of SurA for OMPs is not fully resolved. Aromatic residues have long been considered important, in particular ΦXΦ motifs (where Φ is any aromatic, and X is any residue),^187,190,192,193^ however, peptides lacking aromatic residues also bind SurA,^190,192^ and there are differing numbers of SurA binding sites across different OMPs.^194^ This leaves a key unanswered question of how SurA recognizes and distinguishes its OMP clients from other periplasm-resident proteins or if there are some OMPs it never recognizes.

*E. coli* SurA is comprised of three domains: a Core domain (composed of the N- and C- terminal regions of the polypeptide chain), and two peptidyl prolyl isomerases (PPIase) domains, PPIase-1 and PPIase-2 (only PPIase-2 is functional as a prolyl isomerase^195^) (Figure 4a,b).^187^ SurA is intrinsically dynamic^137,163,191,196,197^ and the PPIase-1 domain can either be bound or released from the Core domain, termed the ‘Compact’ and ‘Extended’ states, respectively (Figure 4a,b).^137,187,192^ These dynamic motions are functionally important since cross-linking PPIase-1 to the Core domain results in OMP assembly defects^184^ and decreased binding of OMPs by SurA.^194^ Apo-SurA (devoid of an OMP client) exists predominantly in its Compact state (∼80%) whereas the presence of an OMP shifts this equilibrium toward the Extended state (∼75%).^137,197^ This suggests that while Apo-SurA samples the Extended state, this state is conformationally selected for by OMP binding.

**Figure 4:**
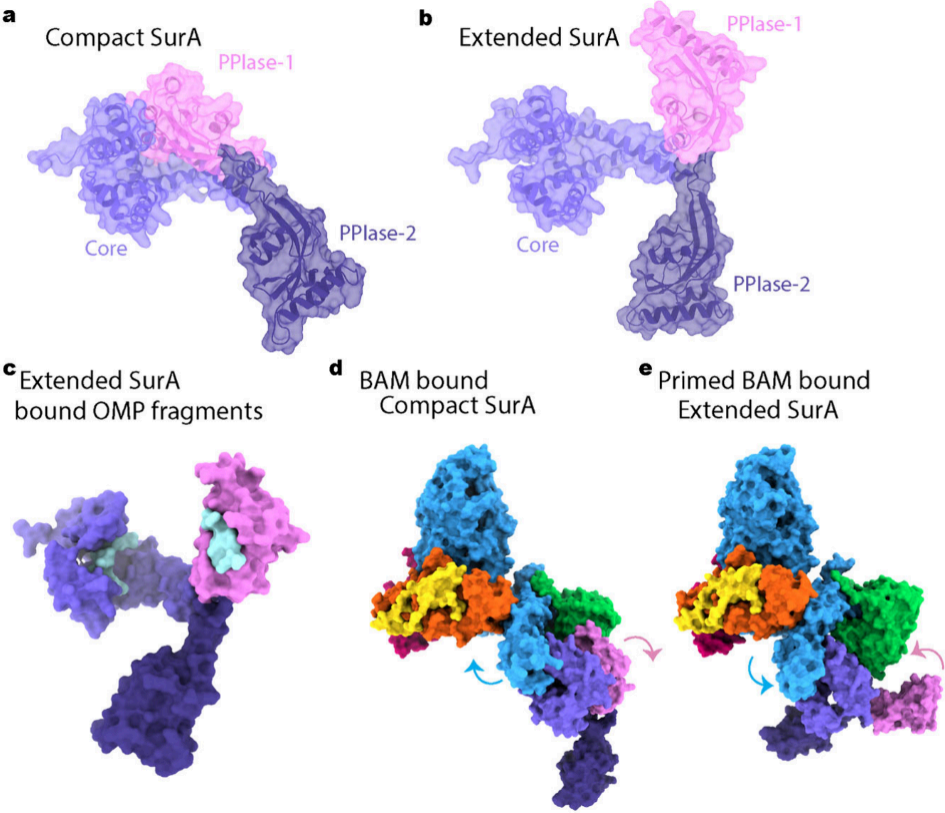
Different conformations of SurA. (a) Compact SurA where PPIase-1 (pink) packs against the Core domain (light purple) (predicted by AlphaFold 3 (AF3) to demonstrate dynamic regions unresolved in the SurA crystal structure (PDB 1M5Y^198^)). (b) Extended SurA where PPIase-1 is released from the Core domain (AF3). (c) Extended SurA bound to OMP fragments (cyan) in its Core and PPIase-1 binding sites (AF3 SurA, aligned with cryoEM structure 8QPV^137^ to show OmpX fragment in Core domain, and peptide WEYIPNV from crystal structure of the PPIase domain 1 (2PV1 ^192^). (d) BAM bound Compact SurA (PDB 8PZ1,^137^ AF3 SurA aligned to demonstrate position of PPIase-2 domain unresolved in cryoEM). (e) Primed BAM bound Extended SurA (PDB 8PZ2,^137^ AF3 Extended SurA aligned to demonstrate position of both PPIase domains unresolved in cryoEM). Arrows in (d and e) shows the motion of POTRA-1 and POTRA-2 that occurs concurrently to the release of PPIase-1 from the SurA core. BamA (blue), BamB (green), BamC (yellow), BamD (orange) and BamE (magenta).

The Extended state of SurA exposes distinct OMP binding sites located in the Core and PPIase-1 domains (cyan regions in Figure 4c)^137,190,192^ as well as a broad region between these two domains that harbors diffuse OMP binding.^194,197^ SurA binding to the unfolded OMP results in expansion of the OMP polypeptide chain^190,194^ and this function is attributed primarily to the Core domain, while the presence of the PPIase domains limits the extent of OMP chain expansion.^190^ This effect on the unfolded OMP has been suggested to help keep the OMP in a folding-ready state and to aid in OMP delivery to BAM.^137,190,192,194,197^

#### 2.4.2 SurA Binding to BAM

Once SurA has bound an OMP and maintained it in a folding competent state, SurA then binds to BAM and delivers its client to initiate its folding. SurA binding to BAM has recently been shown to occur via a key interaction involving its 6-residue long, unstructured N-terminus.^136,137^ Deletion of these six residues results in a loss of OM integrity, that is as severe as deletion of the entire protein on the *E. coli* ‘OMPome’, but results in a less broad σ^E^ response than ΔSurA strains.^137^ This suggests that deletion of these residues has not removed the general chaperoning capability of SurA, but only its ability to deliver OMPs to BAM.

The N-terminal six residues of SurA bind to BamA POTRA-1 via β-augmentation (the formation of a new β-strand using an initial β-strand as a template) and allows SurA to dock to the BAM complex nestling between POTRA-1, POTRA-2 and BamB. CryoEM structures revealed that SurA bound BAM exists in two conformations and the N-terminal β-augmentation interface is maintained in both.^136,137^ In the first, Compact SurA is bound to BAM (Figure 4d). In the second, Extended SurA is bound to ‘primed’ BAM in which the interactions between POTRA-2 and BamD are reorganized, presumably initiating BAM for its catalytic cycle of OMP folding (Figure 4e). The conformational changes observed in both BAM and SurA in this second state suggests a two-way communication wherein SurA’s client binding sites are exposed, and BAM is primed for folding.

### 2.5 β-Signal Engagement with BAM

The initial stages of BAM-mediated OMP folding/assembly are the least well characterized. SurA can bind both Lateral Closed and Lateral Open conformations of BAM via β-augmentation to POTRA-1.^136,137^ Interestingly, addition of the lateral gate locking antibacterial molecule, Darobactin-B^154^ to SurA-bound BAM shifts the equilibrium slightly toward the Lateral Open state^137^ suggesting that SurA-bound BAM favors the Lateral Open conformation.

For the majority of OMPs to enter the OM via BAM, their β-signal is thought to engage with BamA β1 (‘β-signal engagement’). The β-signal is a conserved C-terminal motif (GXXΦXΦ, where Φ is aromatic and X is any amino acid type) found in the majority of *E. coli* OMPs, and which is important for efficient OMP assembly.^55–57^ Although perturbations to the β-signal sequence can prevent efficient OMP folding,^56^ an effect that is amplified by simultaneous deletion of SurA,^199^ several OMPs have been suggested to lack a C-terminal β-signal with a similar motif contained only within internal strands.^199,200^ Intriguingly, cryoEM structures of BAM and SurA in the presence of an OMP do not result in different conformations in BAM, whether that OMP is interacting solely with SurA, or interacting with both SurA and β1 of BamA, suggesting that the presence of SurA does not alter the mechanism of BAM-mediated OMP folding.^137^

AFM experiments, in which OMPs are pulled out of a synthetic bilayer or thereafter relaxed by moving the cantilever allowing them to spontaneously refold, have shown that OMPs both unfold and refold via a single β-hairpin at a time.^138–140^ This lends itself to the idea of a unidirectional model for BAM catalyzed folding, wherein an OMP must fold from its C- to N-terminus, proceeding from the binding of its C-terminal β-signal onto BamA β1. Thus, β-signal engagement is thought of as the initiating step for β-strand production, where BamA β1 serves as the template strand from which the nascent OMP (nOMP) β-barrel nucleates.^57^ However, the route that a β-signal takes from SurA to β1 remains unclear, a problem made even more intriguing by the lack of any observed interaction between the β-signal and SurA.^194^ Instead, BamD has been shown to recognize OMP β-signals *in vivo* and, while the exact role of this interaction remains vague, it is well-established as essential for bacterial viability,^174–176^ hence its integral role is expected to involve substrate recognition.^128,143,177–179^ However, the exact nature and extent of the interaction between BamD and the β-signal is unclear and whether the β-signal interacts with any of the POTRA domains or other BAM lipoproteins prior to and/or post interaction with BamD remains unknown.

The role of the periplasmic ring of the BAM complex after β-signal engagement with BamA β1 is also unclear. Early stage folding intermediates of LptD variants (LptD^Y721D^) were found stalled on BAM while interacting with BamD,^201,202^ and inhibition of such interactions has been shown to cause defects in OMP assembly and OM integrity.^174^ Intriguingly, an internal signal sequence like that of the β-signal, was recently discovered to promote efficient OMP assembly via interactions with BamD prior to β-signal engagement, with initial β-strand formation suggested to begin in the periplasm.^203^ Defined as a nine-residue consensus sequence that can be found 5 strands before the C-terminal β-strand, the internal signal sequence consists of ΦXXXXX­[Ω/Φ]­X­[Ω/Φ] (Φ: Aromatic, X: Any, Ω: Hydrophobic) and is complementary in function to the β-signal. Despite not being essential for bacterial viability, ablation of this interaction did lead to some loss of membrane integrity.^203^

### 2.6 BAM Folding Intermediates

Following β-signal engagement at BamA β1, nOMP folding is thought to occur in a sequential manner from C- to N-terminus. Alongside the aforementioned AFM experiments, individual deletion of the BamA substrate’s (BamA^S^) extracellular loops also supports a unidirectional folding mechanism.^138–140^ This was shown using a series of experiments, including periplasmic degradation, urea extraction, and *in vivo* cross-linking experiments, wherein deletion of the extracellular loops toward the C-terminus of BamA^S^ had a greater impact on OMP folding and membrane integration than deletion of those toward the N-termini, implicating the C-terminal strands of the BamA client as being more important than those at its N-terminus for successful folding and membrane insertion.^141^

The structures of stalled nOMP folding intermediates on BAM have been solved using cryoEM by several research groups, each using cross-linking of the client OMP β-signal to BamA-β1 to trap the complex.^137,141,142,161^ With the nOMP tethered to BamA, a new ‘Wide-Open’ conformation was observed in all stages of folding (from Early and Late to Release) involving sequentially more and more folded β-strands, where the distance between β1 and β16 is even greater than in the Lateral Open state (Figure 3c).^137,142^ Although little additional movement is witnessed in the remainder of the BAM complex, strands adjacent to the β-seam in BamA (β1-β6) appear to be even more flexible than previously thought, further highlighting the unique nature of the BamA barrel. When exactly BamA may shift conformations during the physical assembly of an OMP remains ambiguous, what is clear is that dynamic movement of the BAM complex is integral to its function.

#### 2.6.1 Early Stalled Intermediates

A generalized strategy to observe OMP intermediates caught in the act of folding on BAM has been to lock the OMP β-signal engagement site on BAM via disulfide trapping, ensuring that the nOMP-BamA complex cannot be released. Fenn and co-workers formed a disulfide bond between the C-terminal β8 strand of OmpX and β1 of BamA to capture an early stage folding intermediate referred to as the ‘Handover’ complex, which resulted in the three C-terminal β-strands of OmpX being resolved via cryoEM on BAM (Figure 5d – PDB:8QPV^137^). This structure revealed that while the nOMP is being folded from its C-terminus, SurA (to which the OmpX sequence was concatenated) remains in complex with the unfolded N-terminal region of the OMP. The same overall conformation of BAM-SurA predominates as was observed in the absence of OmpX, wherein BAM is in its ‘primed’ state and SurA is in its Extended state, but now the OMP has begun folding on BAM and the lateral gate is Wide-Open.^137^ Although the use of a concatenated SurA:OmpX construct was utilized in this study, the possibility that SurA may be bound throughout the folding process to keep the unfolded N-terminal end of the folding OMP in a folding competent state, prevent its aggregation, and stop BAM from getting jammed appears plausible.

**Figure 5:**
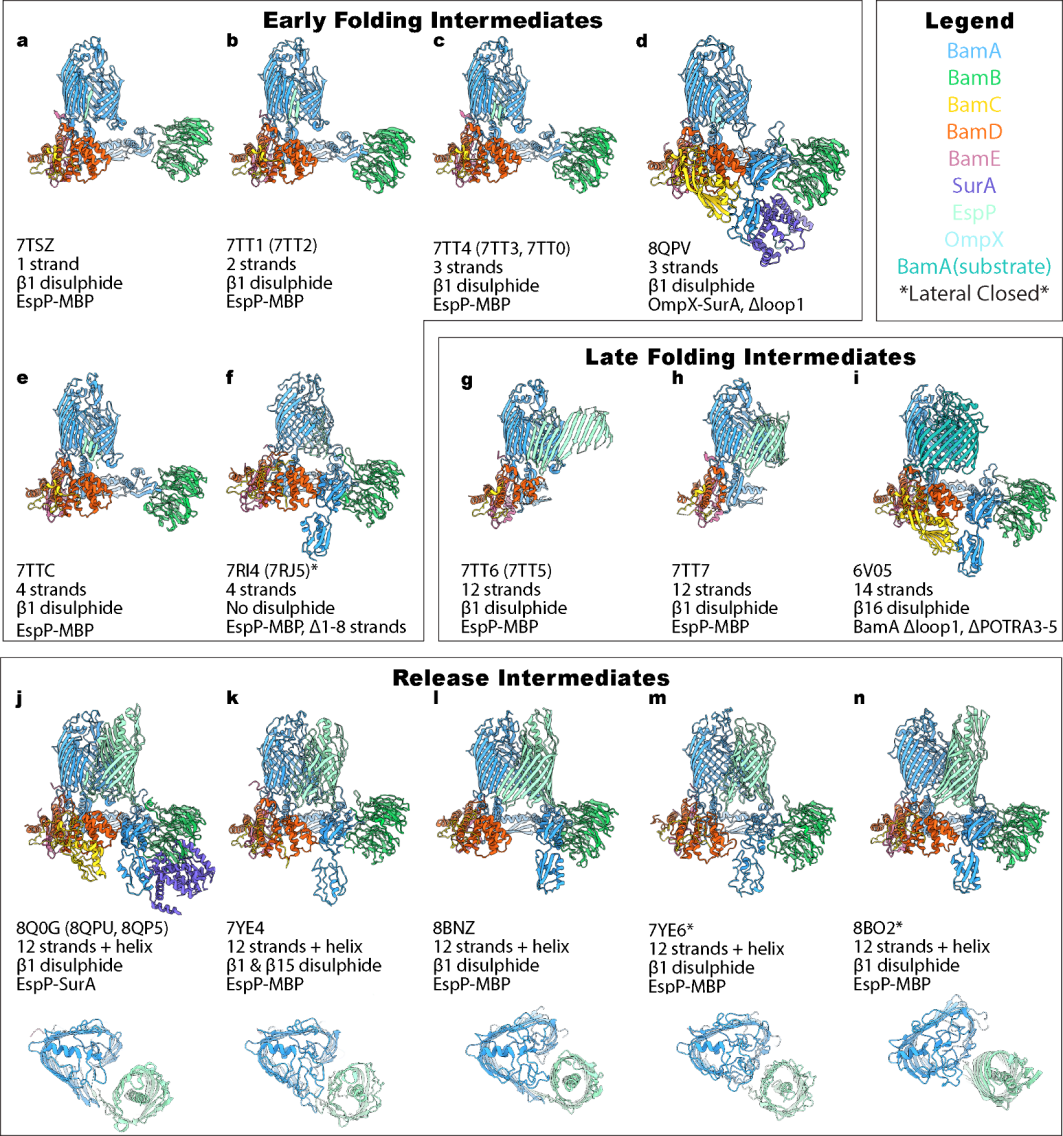
BAM folding intermediates solved by cryoEM. All structures of the BAM complex solved with a substrate bound with PDB code indicated and ordered according to number of strands modeled in the PDB structure (where a similar structure with the same number of β-strands is available the highest resolution structure was chosen for display and the alternative PDB codes are indicated in brackets). Trapping mechanism of the intermediate is displayed demonstrating that all structures have required at least two trapping strategies. All structures were aligned in ChimeraX to the BamA highest resolution Lateral Open structure (9CNW^144^). Top views of the BamA barrel and hybrid EspP barrel are shown for the Release Intermediates to highlight the ‘B-shape’. See for global resolutions and details of membrane mimic used for structure determination.

Alongside OmpX, EspP is the only other OMP witnessed at an ‘early’ stage of folding. EspP can be trapped in an incomplete folding state by replacement of its N-terminal extracellular domain with maltose binding protein (MBP), which rapidly folds in the periplasm effectively ‘jamming’ BAM as the domain is too large for translocation across the OM via BamA.^204^ This approach, coupled with intermolecular cysteine cross-linking between BamA and EspP, identified multiple interaction sites of EspP with BamA, forming a stable antiparallel interaction between the β-signal of EspP and β1 of BamA.^204^ Further work by the same group incorporated the construct into a nanodisc utilizing styrene-maleic acid (SMA) to directly solubilize BAM:EspP along with lipids from the bacterial OM (Figure 5a,b,c,e – PDB:7TSZ/7TT1/7TT4/7TTC^161^). A cysteine cross-link was incorporated between BamA β1 and the nOMP β-signal to stabilize the complex, allowing structures to be solved via cryoEM with four EspP β-strands resolved (β12–9) (Figure 5e – PDB:7TTC^161^) and additional density observed within the membrane. A similar structure was also seen in MSP nanodiscs containing native lipids in which the same four EspP strands (β12–9) were observed attached to BamA, but with additional density underneath the BamA barrel (Figure 5f – PDB:7RI4 ^156^). It should be noted that the latter structure by Wu and colleagues did not use disulfide trapping and found the POTRA domains of BAM adopted a Lateral Closed BAM conformation, within which EspP β12 and β9 interact with β1 and β16 of BamA, respectively to form a super barrel. As a result of this novel structure, the Lateral Wide-Open conformation observed in other stalled cryoEM structures is not found. The formation of this super barrel, and the positioning of the periplasmic ring in the Lateral Closed state is unique among the intermediates identified and highlights the lack of understanding regarding when the transition between Lateral Open/Closed/Wide-Open occurs. Moreover, the conformational differences within the two structures highlights how experimental conditions (such as disulfide traps and specific nanodisc polymers or detergents) may significantly influence intermediate structures, and thus caution is advised when comparing structures across different studies.

#### 2.6.2 Late Stalled Intermediates

Research into late-stage BAM folding intermediates has primarily focused on three diverse nOMP substrates: EspP (12 β-strands) which contains an internal α-helix, BamA (16 β-strands) which has a large internal loop, and LptD (26 β-strands) which folds around the lipoprotein LptE. The structure of the first late folding intermediate was BAM folding BamA^S^. In this seminal study by Tomasek et al.^141^ BamA^S^ had POTRAs-3–5 deleted to prevent the substrate from forming mature BAM complexes and extracellular loop 1 (ΔEL1) of BamA was also missing, stalling BamA^S^ on BAM. The BAM:BamA^S^ complex was also stabilized via disulfide trapping of the BamA^S^ β-signal to β1 of BAM (Figure 5i – PDB: 6V05 ^141^). That the disulfide cross-link did not perturb this structure was validated by switching the disulfide to β16 of BamA^S^-and β1 of BAM and demonstrating, at least in low resolution cryoEM, that the same structure was generated.

As nascent β-strands begin to nucleate from the β-signal engagement site, the nOMP will spontaneously turn back toward itself and BAM to enable folding to continue by forming additional β-strands. Although the mechanism for this action is unclear, it has been proposed to be driven by the elastic force of the membrane (Figure 5g – PDB: 7TT6 ^161^). This eventually leads to the formation of a common ‘B-shaped’ intermediate (observed from below/above the membrane plane), wherein the N-terminus of the nOMP comes into proximity of the β-signal engagement site (Figure 5j-n). Consistent with this, cross-linking between BamA and stalled EspP showed relatively weak and heterogeneous contacts between the outward facing surface of BamA β15/β16 and β1 of EspP.^204^ Slight differences have been observed at the β-signal engagement site (which serves as the backbone of the ‘B-shape’) for BAM:BamA^S^ (Figure 5i – PDB: 6V05 ^156^) and BAM:EspP (Figure 5h – PDB:7TT7 ^161^) intermediates, thought to be due to the increased flexibility of the BamA^S^ barrel.^141,161^ Regardless, the overall architecture of all solved late-stage intermediates retains an approximate ‘B-shape’, with this state consistently witnessed by several independent researchers, through both physical and computational means.^137,142,205^

The ‘rolled out’ intermediate of EspP (Figure 5g – PDB: 7TT6 ^161^), was discovered following extensive refinement of the BAM-EspP­(β12–9) intermediate (discussed in Section 2.6.1, Figure 5e – PDB: 7TTC^161^) and allowed visualization of the remaining EspP strands (β8–1).^161^ This revealed multiple folding intermediates in which the EspP barrel is completely ‘rolled out’ into the membrane, with β1 unpaired, but curling back toward BAM. The authors suggested that the elastic tension of the OM exerts a compressive force that directs the extended EspP sheets back toward the BamA barrel, allowing EspP folding to complete. While the membrane may play a role in ensuring that nOMPs circularize during the folding process, it remains unclear whether the ‘rolled out’ intermediate witnessed by Doyle and co-workers is part of a canonical folding mechanism, especially given the large amount of membrane space required, an extremely limited feature of the *E. coli* OM.

Deletion of EL4 within the sequence of LptD (LptD^Δ330–352^/LptD^4213^) has also resulted in the accumulation of a late stage folding intermediate on the BAM complex,^150,206^ with a similar outcome found via deletion of a single residue at the start of EL4 (LptD^Δ330^).^145^ Employing the functionalized amino acid, para-benzoyl phenylalanine (pBPA), cross-links were found between LptD and sites throughout the inner lumen of the BamA barrel. Similar interactions were witnessed through different cross-linking strategies, implying that LptD folding is catalyzed by the BamA lumen, which may function as an Anfinsen cage for substrate assembly.^201,207^ The observation of similar folding intermediates with BamA^S^ (16 β-strands) with all its ELs intact, as well as direct contacts between residues S439, K610 and N666 in the lumen of BamA with OmpF (16 β-strands) and LamB (18 β-strands), suggests that these contacts are not an anomaly based upon the large size of LptD alone.^145^ Whether this lumen-catalyzed mechanism is unique to LptD and large OMPs, common throughout OMP assembly, or triggered by certain environmental stimuli remains unclear, with clarification hindered further by a lack of atomic resolution structural information currently available.

### 2.7 OMP Release

As hybrid-barrel intermediates are already stable membrane structures, and there is no ATP or proton motive force at the OM at BAM, the release of a fully folded nOMP from the BAM complex must be energetically favorable for it to proceed. Using numerous cross-linking studies in tandem with structural characterization of folding intermediates, a probable mechanism for OMP release from BAM has been proposed,^137,142,161^ with the release mechanism of EspP from BAM being structurally elucidated in detail by exploiting innovative disulfide trapping and cryoEM.^142^ In this model, the release of EspP β12 (its C-terminal β-signal containing strand) from β1 of BamA is facilitated through preferential H-bonding between EspP β12 and EspP β1, in a mechanism assisted by the C-terminal residue of EspP (EspP^R1297^) and the C-terminal kink in β16 of BamA.^142,170^ Four sequential release intermediate structures were solved by Shen and colleagues, each with a novel disulfide bond (or two) trapping the state, and an increasing number of H-bonds present between EspP β1 and EspP β12. The ‘Fully Open’ (Figure 5k - PDB:7YE4 ^142^), ‘Ready-to-Close’ (Figure 5l - PDB:8BNZ^142^), ‘Semi-Closed’ (Figure 5m - PDB:7YE6 ^142^), and ‘Fully Closed’ (Figure 5n - PDB:8B02 ^142^) conformations contain one, three, eight and twelve H-bonds, respectively, at the β-seam of EspP, representing different stages of closing of its β-barrel.^142^ As multiple intermediates with an increasing number of H-bonds were captured during closure of the EspP barrel, a processive zipper like mechanism consistent with the gradual exchange of hydrogen bonds appears the most likely molecular pathway enabling barrel release, as opposed to a single step release mechanism.

The ‘Fully Open’ (Figure 5k - PDB:7YE4 ^142^) intermediate structure is also in close agreement with a late-stage intermediate structure of EspP folding on BAM determined by Fenn et al., (Figure 5j – PDB:8Q0G^137^), highlighting the reproducibility of such a state. However, the disulfide trap in the latter work is actually the same as that in the ‘Fully Closed’ structure (Figure 5n - PDB:8B02 ^142^) (). Despite both being trapped *in vivo*, the conformational differences are likely a result of reconstitution into different membrane environments (detergent^137^ vs nanodiscs^142^). Hence, caution should be taken when comparing atomic resolution detail across structures gathered in different membrane mimetics, and future work should strive to utilize physiologically relevant membrane conditions as much as possible.

Alongside this zipper-like mechanism within the membrane, a processive rotation of the periplasmic ring of BamA’s POTRA domains was also observed in the structures of Shen et al. as the EspP barrel completes folding and is gradually released.^142^ It should be noted, however, that within the “Ready-to-Close” structure (Figure 5l - PDB:8BNZ^142^), where β-signal engagement remains intact and multiple H-bonds are also present between β1-β12 of EspP, BamD is no longer in contact with POTRA-1 or POTRA-2. If this structure recapitulates the mechanism of substrate release *in vivo* (i.e., without trapping), the findings suggest that BamD disrupts its interactions with the POTRA domains and moves away from its contacts with BamA, before movement of the POTRA domains then allows the interaction network to be reformed. Further work is required to understand whether this proposed mechanism is correct. However, this seems more unlikely than a “push-pull” like movement facilitated by the Lateral Gating movement of the POTRA domains. How the gradual transition from Lateral Open to Lateral Closed states applies to other substrates remains an open question, including if, and how, BAM might tailor release mechanisms based on the specific OMP being assembled.

Our understanding of the mechanism for BAM assisted OMP assembly has developed dramatically over the past ten years, yet while a general framework for this multistep process has been established, the intricate molecular details of several events remain lacking. This is particularly evident when it comes to the delivery of an unfolded OMP from SurA to β1 of BamA, where very little is currently understood. Future work involving clever design of kinetic traps to capture other intermediate states will be needed to answer these points.

## 3 BAM Lipoproteins

Whereas BamA is strongly conserved across diderm bacteria, BAM’s lipoproteins vary much more significantly across bacterial species,^208^ with their more limited conservation likely due to a lesser (or more varied) importance in the OM assembly process. Indeed, a significant amount of work has been carried out analyzing *bamB*, *bamC*, *bamD*, and *bamE* knockouts in multiple different combinations and conditions to try to identify roles for BAM lipoproteins, which have resulted in a variety of phenotypes, ranging from minor changes in OM assembly to serious growth defects.^97,152,209,210^ The consensus within the field is that BamD is essential for BAM function alongside BamA, while BamB, BamC and BamE all share a redundant role that primarily involves the correct orientation of BamAD. Here we collate current information pertaining to genetic knockouts of the BAM lipoproteins to provide a holistic, yet simplified overview of the phenotypes within *E. coli* (see also ).

### 3.1 BamB (YfgL)

Formerly known as YfgL, BamB was first observed in 2005 as a contributor to bacterial virulence^211^ before later being identified as a lipoprotein capable of direct interactions with BamA.^132,212–214^ Interacting primarily with POTRA-3, BamB sits alone on the opposing side of the POTRA domains to the other three lipoproteins (Figure 2).^143,215,216^ Structurally, BamB is a β-propeller protein that shares homology to the WD40 family in eukaryotes, well established as facilitators of protein–protein interactions within complex assemblies.^217^ So it came as little surprise when BamB was found to interact with BamA, as well as BamB *in trans* to form ‘folding precincts’, defined as the arrangement of several BAM complexes mediated by BamB-BamB interactions in proximal BAM complexes in the OM.^97^

The study of Δ*bamB* strains has revealed OM permeability defects, likely a result of impaired OMP assembly. Thewasano and colleagues have reported the trimerization of OmpC, OmpF and LamB to be significantly hindered in Δ*bamB* strains, in addition to a reliance on the presence of BamB for sufficient assembly of OMPs with greater than sixteen β-strands.^97,209^ Alongside its roles in creating large protein assemblies, BamB has recently been implicated alongside different molecular chaperones where it may serve as an interaction platform, whether that be via interactions with SurA during the delivery of nOMPs to BAM,^137^ or in the chaperone-usher (CU) pathway during P pili biogenesis.^218^ Indeed, a SurA mutant (S220A), which exists only in the Extended conformation (Figure 4b), overcomes a ΔBamB phenotype, supporting a role of BamB in modulating the dynamics of SurA.^185^

### 3.2 BamC (NlpB)

BamC (previously NlpB) remains the most mysterious of the BAM lipoproteins with little understood regarding its function, and its inherent flexibility in the BAM complex making structural elucidation challenging.^219–221^ Within BAM, BamC interacts primarily via its N-terminal ‘lasso’ which coils around BamD, although contacts have also been reported between BamC and BamE and POTRA-1.^73,143,152^ A role of BamC in stabilizing BamAD interactions alongside BamE has also been proposed, although conclusive evidence is yet to be found to support this function.^73^ As well as its lasso domain, BamC also contains two C-terminal helix grip domains, the first is thought to weakly interact with POTRA-2, although this has only been observed transiently in the absence of BamB.^73^ The positioning of the second helix grip domain in BAM remained elusive due to its dynamic properties, with the structure only observed in the protein in isolation.^73,136,222^ However, in a recent cryoEM structure of extended SurA bound to BAM, the C-terminal domain of BamC was observed interacting with POTRA-2, which the authors suggest implicates BamC in a coordinated function in OMP biogenesis alongside SurA, with the two proteins proposed to control substrate insertion through conformational cycling.^136^

Δ*bamC* strains appear to have the least severe impact of all lipoprotein deletions, with the absence of BamC shown to have little impact on bacterial viability, OM permeability, and OMP assembly compared with the other BAM-associated lipoproteins.^132,209,219,223^ Deciphering the functional role of BamC is complicated further by the reported surface exposure of its C-terminal domain discovered via immunofluorescence microscopy.^224^ Yet to be confirmed via any other means, this observation suggests that BamC can traverse the OM, although the functional implications of this remain unclear.

### 3.3 BamD (YfiO)

Within the BAM complex only BamA and BamD (previously YfiO) are essential for cell viability.^212,219,225^ BamD consists of five tetratricopeptide repeat (TPR) domains, with the binding of TPR3/4 to POTRA-5 integral for BAM activity with perturbations in this interaction causing severe cell phenotypes.^73,226^ Recent pulsed electron–electron double resonance (PELDOR), has suggested that this interaction site may drive the transition from Lateral Closed to Lateral Open upon association of BamCDE with BamA.^227^ Further contacts are also formed between BamD and BamA at Turn-2, POTRA-1 and POTRA-2, which complete the periplasmic ring structure.^73^ A recent study utilizing *in vivo* FRET found that the BamAD interface is perturbed by peptides mimicking the BamD sequence in a dose dependent manner, opening the door to the potential for therapeutic targetting.^228^

The primary function of BamD is thought to involve recognition of nOMPs via their β-signals.^172–174^ Cross-linking experiments, both *in vivo* and *in vitro*, suggests that recognition of nOMPs via BamD is quickly followed by β-signal engagement on BamA that initiates OMP folding and assembly.^150,201,229^ Furthermore, BamD has recently been proposed as capable of commencing the folding process of OMPs via a second, internal signal sequence located five β-strands away from the C-terminal β-signal.^203^ However, as this sequence was found to not be essential for bacterial viability, its role within the BAM assembly mechanism remains unclear.

Multiple single point mutations in BamA (E470K, A496P, A499S) thought to facilitate improved nOMP engagement, have been found to bypass the necessity for BamD for bacterial viability and OMP assembly.^210,230^ While the mechanism for this is unclear, Hart and colleagues suggest that this demonstrates a role for BamD in regulating BamA activity, rather than a direct catalytic role in OMP assembly.^210^ Likewise, mutations in RcsF (G117R) that are thought to destabilize BamA jamming have recently also been discovered to bypass the necessity for BamD.^231^ Taken together, current literature supports a model in which BamD ensures the efficient engagement of nOMPs by BamA allowing for subsequent OMP assembly, thus making BamD essential to cell viability. Conversely, a recent study utilizing a sophisticated *in vitro* spheroplast based folding assay, found BamADE to be the minimal functional form of the BAM complex capable of OMP assembly even with BamA^E470K^ present.^232^

### 3.4 BamE (smpA)

BamE (previously smpA) was the last of the BAM components to be identified, due to its small size (∼12 kDa) and lack of obvious phenotypes in knockout strains.^225,233^ BamE interacts with POTRA-5 of BamA and BamD, stabilizing the BamAD complex along with BamC.^143,152^ Genetic experiments suggest that the individual roles of BamC and BamE are not completely redundant, with BamE also thought to modulate the conformation of BamA.^223^ This theory was reaffirmed through neutron reflectometry experiments where interactions between BamE and POTRA-5 triggered a conformational change in POTRAs-3–5, with the domains moving away from the membrane.^234^ A tripartite interaction network between BamADE has now been proposed as important for BAM function.^235^

The loss of BamE reduces OMP assembly regardless of barrel size, while loss of the often-compared BamC has little to no impact.^209^ One explanation could be an interaction of BamE and SurA. AlphaFold2 (AF2) and hydrogen–deuterium exchange (HDX) suggest that BamE interacts with PPIase-2 of SurA,^162,163^ but this interaction was not observed in any of the cryoEM structures of BAM-SurA reported to date.^136,137^ Deletion of PPIase-2 has drastic effects on the rates of *in vitro* BAM catalyzed folding rates^163^ and yet despite rigorous studies using NMR no OMP binding has been attributed to this domain.^190,236^ An interaction between SurA and BamE is intriguing but it is either a weak, transient interaction or formed only at a specific point during an OMP folding cycle. BamE has also been found to form dimers, with BAM complexes consisting of BamABCD­(E)_2_ also observed via native mass spectrometry, although any functional significance of this observation remains unclear.^173,237–239^

Much remains unknown regarding the BAM lipoproteins, particularly in understanding precise roles within the process of OMP assembly. Redundant functionalities and the ability of bacteria to sustain OMP folding in their absence, often by triggering stress responses, have made understanding specific roles a tall order, with phenotypes witnessed from genetic deletions difficult to interpret. What is clear is that there is a complex interplay of the lipoproteins with each other, with BamA, and even with the nOMP, which together optimize folding of OMPs into the OM.^232^

## 4 Targeting the BAM Complex

The essential nature of the BAM complex for the construction and maintenance of the OM of diderm bacteria means it has long been considered an attractive target for antimicrobials. Studies have targeted BAM with antibodies, peptidomimetics and small molecules with varying levels of success, but as yet no new therapeutic has made it to market.^153–155,160,240–247^ However, several of the inhibitors generated have aided our understanding of the mechanism(s) of action of BAM in OMP folding and membrane insertion. There are several major challenges in targeting BAM to generate new antibiotics. First, any molecule must be able to pass through the polysaccharide layer of LPS to reach BAM in the bilayer, and then only a small surface of BAM is exposed to the extracellular environment for binding. Molecules that target regions of BamA in the periplasm and/or BamB-E must penetrate the OM to reach their targets. Finally, the dynamic nature of BAM represents another challenge for inhibitor design. The modes of action of some of these inhibitors have been demonstrated. The majority target BamA, and broadly fall into 3 classes: (i) β-signal peptide mimics, (ii) antibodies/nanobodies that bind the extracellular loops, and (iii) peptides that bind to the BamA barrel (Figure 6, see refs 248, 249 for recent reviews).

**Figure 6:**
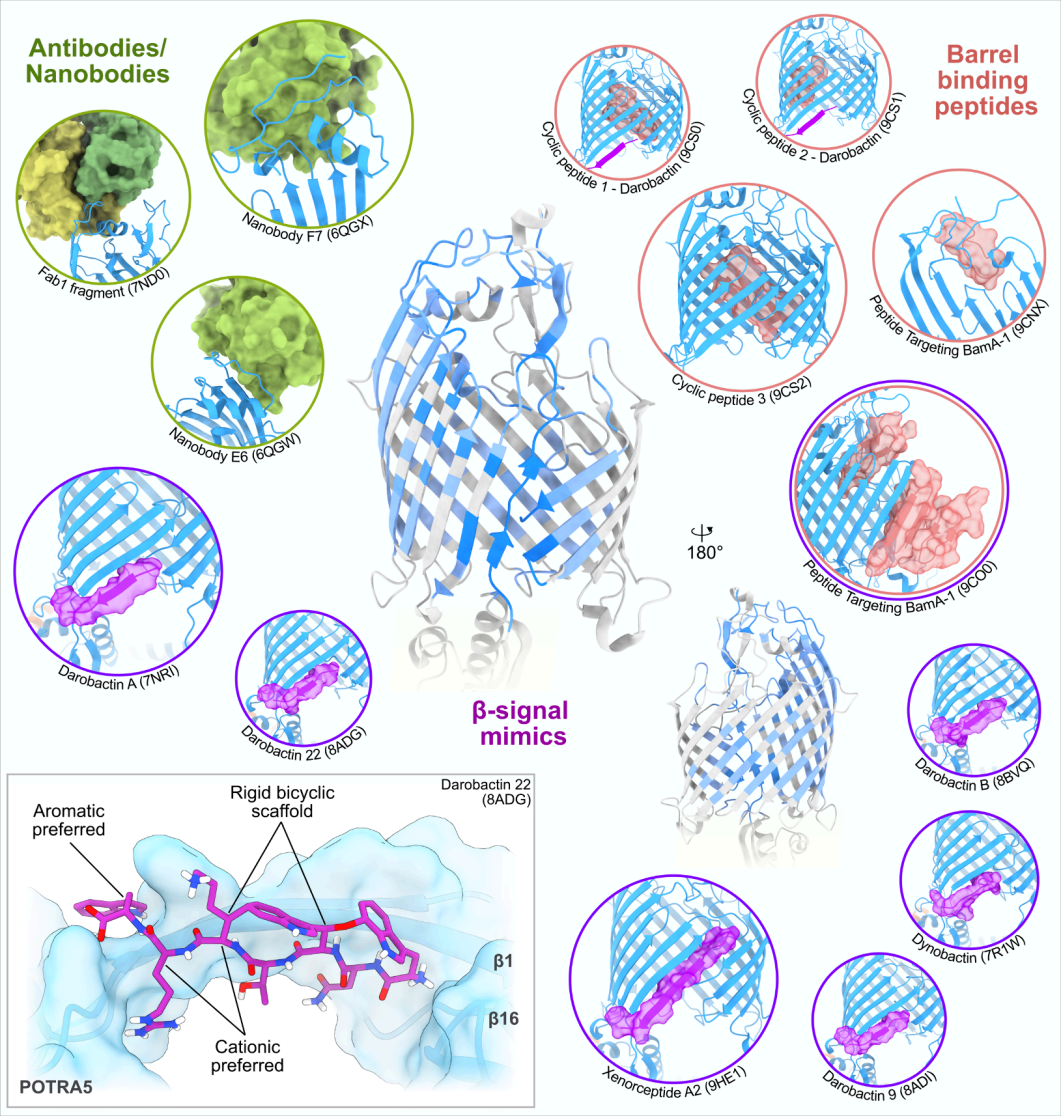
Structurally resolved inhibitors of BAM. BAM inhibitors broadly cluster as nanobodies/antibodies that bind extracellular loops (green), β-signal mimics (purple) and BamA barrel binding peptides (orange). Central BamA barrel structure is colored by interaction frequency with inhibitor molecules (gray: no interactions, light-blue to dark-blue: increasing number of interactions), highlighting that much of the surface of BamA is a potential inhibitor target. Additional inhibitors have been characterized as binding to BamA, but their specific binding sites were not identified. Inset: Close-up of BamA:Darobactin-22 interaction, highlighting key features of the darobactin class.

The most well characterized and chemically optimized class of inhibitors are the β-signal mimics. Intuitively BamA-β1 is an ideal target on BAM to inhibit OMP folding because initiation of membrane folding/insertion occurs via β-signal engagement at BamA-β1 and the interaction is maintained throughout the folding process. In fact, a search for antibiotics led to the identification of an antimicrobial, darobactin, from *Photorhabdus khanii*, a nematode symbiont released by nematodes upon insect larvae invasion. *P. khanji* releases antimicrobials to fend off environmental microorganisms,^242^ including darobactin. Studies revealed that darobactins bind to BamA-β1 by forming a β-strand and effectively mimicking the β-signal, consequently darobactin is unable to bind BAM after it has already engaged with an OMP at β1.^250^ Structural studies of the BAM complex in the presence of darobactin or darobactin-like molecules reveal a stabilization of the BAM complex in its Lateral Closed conformation (Figure 6, purple).^154,155,160,242,247^

The originally identified darobactin, darobactin-A, is a macrocyclic heptapeptide with the sequence WNWSKSF (). It features two rings formed between the two Trp residues (an alkyl-aryl ether bond) and between Trp-3 and the Lys-6 (a carbon-carbon bond). The rigidity these cross-links provide to darobactins give them the ability to mimic the β-signal and facilitates a higher affinity binding to BamA compared to linear peptides lacking these additional linkages. The discovery of darobactin-B (WNWTKRF) yielded a compound with a 4-fold increase in potency compared with darobactin-A. A variety of darobactins have now been discovered/generated by systematically changing or improving the sequence (see Dutta et al 2024 ^251^ for a comprehensive list of peptides and their minimum inhibitory concentrations against different bacterial strains). Although mutating the sequence changed the potency, different darobactins exhibited varying inhibitory effects against different bacterial strains and species. This makes it difficult to draw conclusions on the best darobactin sequence which may reflect variation in the outer membrane or BAM of these strains/species.^251^

The nontrivial nature of novel antibacterial design/discovery is highlighted in the discovery of dynobactins by a computational analysis of biosynthetic gene clusters (BGCs) distantly related to the darobactin BGC. The resulting darobactin-like molecule is a decapeptide (WNSNVHSYRF, ) again with macrocyclic rings (a carbon-carbon bond between the Trp-1 and Asn-4 and a carbon-nitrogen bond between His-6 and Tyr-8) that shows 2-fold greater potency *in vitro* relative to darobactin-A but is 4-fold less potent against *E. coli.* This effect results from a decreased ability of dynobactin to cross the OM. Dynobactin shows good solubility in water and minimal cytotoxicity to mammalian cells, highlighting the potential of antibiotic development from this compound.^155,252^ Methods for total synthesis for both darobactin and dynobactin have been demonstrated and thus both represent good starting points for chemical optimization.^253,254^ Many related BGCs have been identified which potentially produce new natural BamA targeting antimicrobials, paving the way for the development of new compounds against BAM.^155^

Darobactins were rapidly employed as powerful tools to study the conformational ensemble of BAM. For example, the addition of darobactin to BAM-SurA complexes showed that SurA can bind to both Lateral Open and Lateral Closed BAM without altering the BAM-SurA binding interface.^136,137^ The addition of darobactin-B to intact *E.coli* cells was also shown to modulate the BAM conformational ensemble *in vivo*.^154^ Other inhibitors have been used to stabilize a given BAM conformation, and used to demonstrate that inhibiting the dynamics of BAM prevents OMP folding. A nanobody and antibody binding^153,243,244^ to BAM extracellular loops have been shown to stabilize the Lateral Open conformation, while a different nanobody and a cyclic peptide have been discovered that stabilize the Lateral Closed conformation.^144^ Several cyclic peptides have been identified that bind in the lumen of the BamA barrel with stabilization of the Lateral Closed or Lateral Open states again observed (`̀̀̀̀̀Figure 6, green and orange).^144,241^ Intriguingly the peptide that stabilizes the Lateral Open state has been shown to also bind BamA-β1 and form β strands that mimic a folding OMP.^144^ A recent preprint identifies a new protein antibiotic, L-type pyocin, that combines features of the loop binding antibodies with the β-signal mimics into one molecule which initially binds the BamA loops and then releases a C-terminal peptide to bind BamA β1. Interestingly, these pyocins are released by *P. aeruginosa* to eliminate closely related competitor strains/species. These represent an exciting new avenue for engineering proteins that target the BAM complex.

One of the major reasons for a lack of breakthrough in the development of new antimicrobials is that bacteria rapidly develop resistance to inhibition. This has been demonstrated for both the antibodies and small molecules that target BAM.^242,244^ Interestingly these mutations are often located at sites far away from the binding sites of the inhibitors. For example, several resistance mutations to antibody inhibition were located either at the back of the BamA barrel or on POTRA-4 and not in the antibody binding site on BamA extracellular loop 4, suggesting that a better understanding of long-range signaling within BamA and between the different components of the BAM complex is required for the design of new effective antimicrobials against the BAM complex. This also highlights the likely key role of allosteric regulation of BAM during the folding process, which is yet to be fully appreciated and understood. These other inhibitors have shown BamA inhibition but have lacked systematic optimization to improve potency or decrease resistance potential to date.

## 5 BAM Periplasmic Partners

### 5.1 Removal of Defective OMPs from BAM

BAM folds OMPs into the OM, but bacteria need this process to be efficient and controlled to maintain OM integrity and limit the accumulation of unfolded proteins that could aggregate, block BAM and prevent OMP folding. To this end there are several mechanisms by which bacteria can remove defective OMPs that are stalled on BAM and unable complete their folding. The ability to identify, remove, and/or recycle stalled or misfolded OMPs typically involves highly optimized machinery consisting of numerous components all working together, making these processes difficult to study one by one. This further complicates, and make even more impressive, the feats involved in the capturing of OMP folding intermediates *in vivo* that have been necessary to reveal the current understanding of the BAM mechanism.

DegP and Skp operate together to minimize the accumulation of early stage folding intermediates on BAM, playing the roles of protease and sacrificial chaperone, respectively.^225^ Monomeric, disordered Skp molecules reside in the periplasm, whereupon contacting an unfolded OMP they assemble to a trimeric state around the bound OMP client. This OMP binding is mediated by multiple weak transient interactions with nonspecific sequences which enables Skp to bind the broad range of OMP clients. Skp’s multiple weak interactions with OMPs generates a high affinity of Skp for OMP clients (low nanomolar affinity), resulting in Skp-OMP complexes that are highly stable and hence prevent the OMP’s aggregation. These Skp-OMP complexes display lifetimes on the order of hours such that Skp acts as a sacrificial adaptor protein and the full Skp-OMP complex is degraded by DegP, a serine protease that forms cages around its clients before proteolysis occurs. Skp can remove OMPs that have already begun folding on BAM. For example, Skp has been shown to be able to remove LamB with up to six or seven folded strands (out of a possible eighteen) but, when more strands of LamB are folded on BAM, Skp is unable to remove the OMP from its BAM-bound state. OMPs with defective β-signals are also bound by Skp.^199^ Together this suggests that Skp and DegP work together to remove OMPs whose folding has failed early in the folding cycle. A direct Skp-BAM interaction has never been shown, and exactly when and what triggers Skp binding to OMPs that are less capable of folding or stalled on BAM remains unclear.

Direct proteolytic cleavage of OMPs folding on BAM is carried out by the protease BepA, which directly interacts with BAM. Accordingly, BepA can be pulled down with BAM and *in vivo* has been shown to cross-link to BamA, BamC and BamD. Alphafold2 (AF2) predictions suggest that BepA could interact within BAM’s periplasmic ring.^162^ BepA has been shown to degrade late-stage stalled OMPs and hence it acts as a last resort protease.^77^ BepA is regulated by the σ^E^ stress response which monitors the accumulation of unfolded OMPs in the periplasm.,^182^ BepA is expressed when many BAM’s are stalled with misfolded or slow-folding substrates and there is a consequent increase in unfolded OMPs in the periplasm. Thus far, BamA and LptD are the only BepA substrates that have been identified,^77^ and it remains to be seen if any other, or indeed, all late-stage stalled OMP are degraded by BepA. The exact mechanism of proteolytic cleavage of stalled OMPs by BepA is unknown, as is how BepA exclusively recognizes and degrades late-stage stalled OMPs. What happens to a cleaved, late-stage stalled OMP is also unclear, as removing an already membrane integrated and nearly fully folded OMP would be highly unfavorable thermodynamically.

There are two additional zinc metalloproteases linked to regulation of OMP biogenesis, lipoproteins YcaL and LoiP. Like BepA, these proteases are members of the M48 protease family, which is currently poorly understood.,^77^ YcaL has been shown to be capable of degrading early stage folding LptD, but a different population of conformations than are cleaved by DegP.^77^ LoiP cleaves the peptide bond between two phenylalanine residues and there is some evidence it may be able to form a complex with BepA. Why and when these proteases act on folding OMPs is unknown, as is how they might access an OMP folding on BAM given their own membrane localization. Further work is clearly required to understand the precise temporal regulation involved for all these OMP assembly regulators. How these proteins sense which BAM is stalled or folding an OMP at a slower rate is also unclear. Such an understanding would not only enhance our appreciation of OM biogenesis but may also yield other opportunities to generate strategies for developing new antibacterial drugs.

### 5.2 BAM and Protecting the OM

Given the importance of the OM for bacterial viability and the integral role of BAM for generation and maintenance of this barrier, it is not surprising that BAM also plays a role in several protective mechanisms that are upregulated when the OM is damaged or stressed. The σ^E^ stress response monitors the accumulation of unfolded OMPs in the periplasm and its activation induces the expression of the σ^E^ regulon.,^182^ Many of the proteins within the OMP biogenesis pathway are regulated by σ^Ε^. The expression of both SurA and the individual subunits of the BAM complex are upregulated to enable increased OMP folding. DolP, a lipoprotein, has also been shown to be upregulated by σ^Ε^, interacting with BamA and coordinating its efficient folding in a chaperone-like manner. The expression of Skp, DegP and BepA are also all upregulated by σ^E^ activation, to enable removal of misfolded OMPs.^266^ Finally, small noncoding RNA represses the expression of the most abundant OMPs, OmpC, OmpF and OmpA, to reduce OMP flux through the folding pathway.

The Rcs system is a two-component system that detects envelope stress, in particular OM or peptidoglycan damage, and regulates gene expression in response to these stresses. The system is complex and involves several different proteins to relay the signal from the OM into the cytoplasm. The first step in signal transduction under stress conditions requires the OM-integrated protein RcsF to interact with the negative regulator of Rcs, IM-integrated IgaA, relieving Rcs repression and triggering the subsequent signaling cascade. This places RcsF as the crucial sensor of OM stress. RcsF is a lipoprotein that interacts with OMPs, with *in vivo* cross-linking suggesting that it interacts with BamA, OmpC, OmpF, and OmpA. The RcsF interactions differ between the different OMPs. RcsF interacts similarly with OmpC and OmpF in the lumen of their barrels and with BamA in its lumen but using different interaction interfaces. In contrast, OmpA, a smaller 8-stranded OMP, interacts with RcsF weakly via its periplasmic domain. The packaging of RcsF into the lumen of OmpC and OmpF is coordinated during their folding by BAM, although how BAM coordinates simultaneous OMP folding with RcsF packaging remains elusive. RcsF interaction with BamA in the lumen requires BamA to be in the Lateral Closed conformation and it also appears that RcsF remains bound to BamA in the absence of BamC, BamD and BamE. RcsF can be located extracellularly, but the extent of its extracellular exposure and a mechanism for its export is undetermined.

How exactly RcsF senses stress is the subject of debate, with two broad models which are not necessarily mutually exclusive. The first proposal is that that the RcsF molecules that have integrated with OMPs are ‘lost’ to the Rcs system, but RcsF molecules that fail to be integrated with an OMP during folding are the triggers of a signaling cascade. This places BAM in a sensor-like role where changes to the OM leads to changes in the flux of OMPs, which in turn which leads to more RcsF that has not been OMP integrated thereby triggering Rcs signaling. The second model suggests that OMP-RcsF complexes are disrupted by OM defects and disassemble so that RcsF becomes exposed to the periplasm, which in turn initiates the signaling cascade. Both theories appear to require BAM for the funnelling of RcsF to OMPs, placing BAM as an essential factor for the proper functioning of the Rcs system.

Another enigmatic protein is SlyB. This lipoprotein is part of the PhoPQ two component system that responds to low pH, divalent cation shortage, or antimicrobial peptides, which destabilize the OM by shedding LPS which causes lipid flipping and loss of OM asymmetry. The PhoPQ system counteracts this by expressing SlyB which oligomerizes into ring-shaped transmembrane complexes that may encapsulate OMPs only when they are within lipid bilayers in the absence of LPS. This encapsulation stabilizes the OMP as well as preventing the rupture of the OM at these lipid nanodomains. SlyB as an OM guard protein was initially identified in complex with BamA and clearly results in inactive BAM complexes. SlyB is also under σ^Ε^ regulation and protecting OMPs and the OM from rupture clearly confers a selective advantage. However, many questions remain. Exactly how SlyB senses and inserts into the OM, how it selects the small percentage of OMPs it protects, how this prevents the cell from rupture, and what happens to it (and the encapsulated OMPs, including BAM itself) once lipid asymmetry has been restored is unclear.

## 6 Conclusions and Perspectives

Over the past decade substantial progress has been made in developing our understanding of the mechanistic details involved in BAM-mediated folding of an OMP, however several key questions remain unanswered. Some of these are highlighted in Figure 7. SurA has been shown to bind BamA at POTRA-1 through β-augmentation, with or without an OMP present, yet it is not currently understood which is preferential.^136,137^ Does SurA bind BAM in the absence of an OMP *in vivo*, thus priming both itself and BAM to accept an OMP from a second SurA which is bound to an OMP client? Handoff between SurA molecules is consistent with observations that multiple SurA’s can bind a single unfolded OMP.^194^ This suggests that there might be some SurA’s that interact with OMPs as they are released from the IM and some that remain BAM bound at the OM. Perhaps there are successive handoff events between SurA molecules or between SurA and other chaperones occurring across the periplasm, but how SurA and/or the SurA-OMP complexes traverse the periplasm remains unclear. A ‘supercomplex’ has been proposed where a ‘bridge’ across the periplasm between the SEC translocon and BAM is formed. However, how common, stable and the exact IM proteins involved in such a complex remains debated. Equally the role of other chaperones in delivery to BAM is unclear.

**Figure 7:**
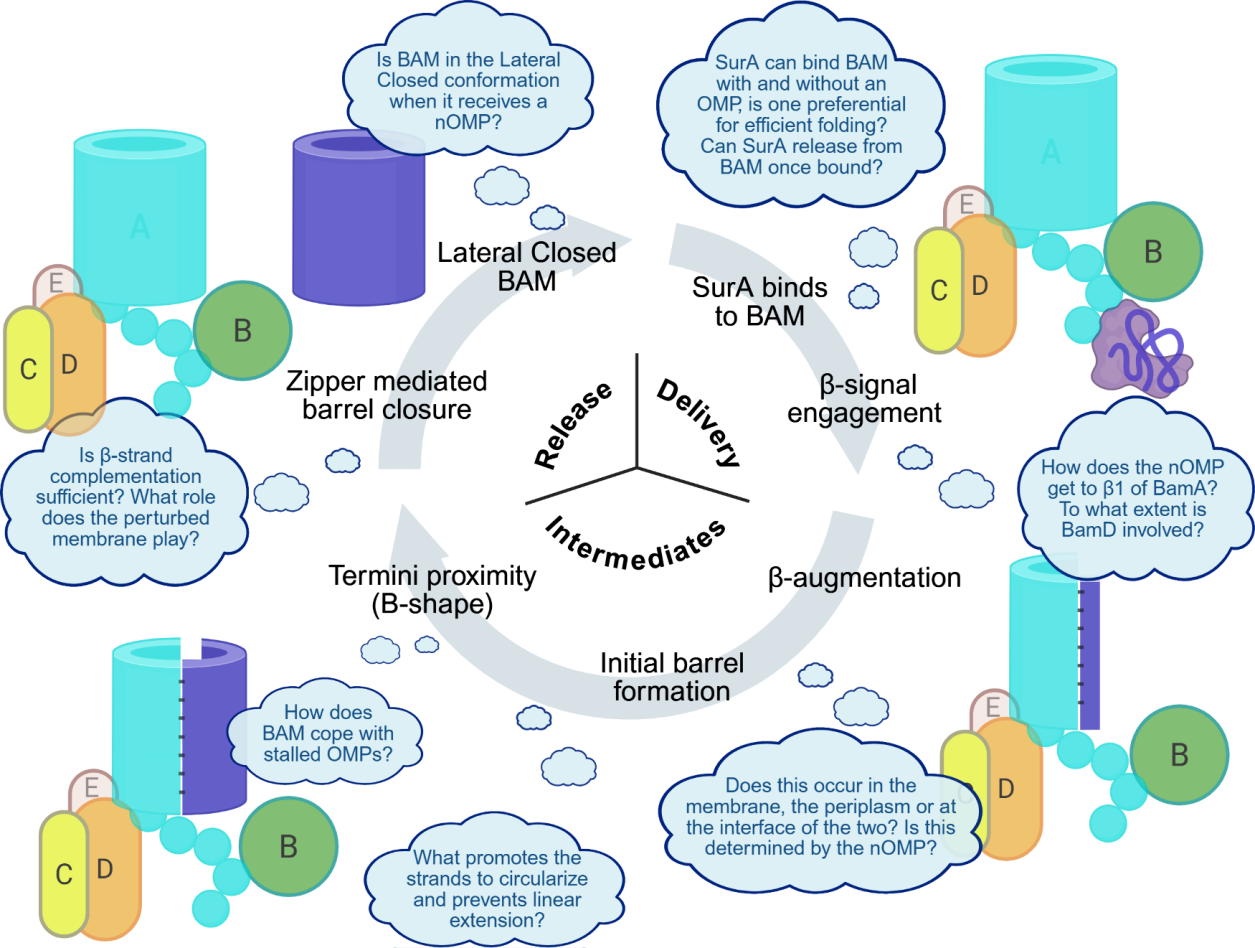
BAM mediated folding. An overview of how BAM folds OMPs, highlighting current understanding and key questions that remain to be answered. Created in BioRender. https://BioRender.com/rimg2ya.

Another major question is how SurA releases an OMP such that it can begin its folding cycle on BAM. SurA does not appear to bind the β-signal,^194^ raising the possibility that this allows SurA to position the OMP such that the β signal is then able to bind to BamD or BamA in BAM. The dynamics of how SurA binds and releases its clients in a manner that they can fold correctly and vectorially from C- to N-terminus on BAM is unknown. When the unfolded OMP is delivered to BAM, SurA can remain bound during β-signal engagement but how it coordinates release of the OMP from its binding sites in a timely manner that correlates with folding on BAM remains a mystery. SurA’s ability to facilitate changes in BamAD interactions^137^ alongside its dynamic motions while POTRA-1 bound^136^ suggests that SurA plays a more involved role in OMP delivery than simply protecting the polypeptide chain from aggregation. It is unclear whether SurA remains bound for the entirety of BAM’s folding cycle or what governs its release from the BAM complex. There are several options that might control its release: perhaps it is released when the SurA has no more OMP bound in its binding sites or maybe a conformational change in BAM during release of an OMP could cause the release of SurA.

Despite β-signal engagement being well established as an important step in BAM-catalyzed OMP folding, how the C-terminal region of the folding nOMP finds β1 of BamA from SurA remains a mystery. Conformational dynamics of both BamC and SurA moving in synergy have been touted to facilitate insertion of an OMP,^136^ but insertion to where exactly? A processive model has previously been discussed wherein the nOMP traverses the POTRA domains through transient interactions before interacting with BamD and then finally arriving at β1 of BamA. However, there is no conclusive evidence for such a model. What is clear is that BamD seems to play a role in this journey by interacting with the folding OMP, although how this is choreographed and its role in OMP delivery to BamA β1 is unknown.

OMPs are generally thought to begin folding via templating from BamA’s first strand (β1) which allows the stepwise addition of β-strands or β-hairpins.^138–140^ Assembly of multiple β-strands within the periplasm has been observed for some (larger) nOMP substrates (LptD and OmpC), coupled with BamD interactions.^150,203^ Does the canonical folding pathway involve periplasmic nOMP folding? Or is this a compensatory mechanism that is uniquely required to fold larger substrates? Regardless, the β-sheets formed in the periplasm would have to somehow make their way into the membrane. A swing mechanism coupled to the Lateral Gating of BamA as well as interactions between the nOMP chain and BamA lumen have both been suggested, however neither model has been definitively proven.^175,204^ As the OMP continues to fold within the membrane, intermediate structures have shown how the nucleating β-strands eventually turn back toward BAM to form the expected barrel-like shape.^137,142,161^ However, the question as to why this happens remains unanswered. Elastic tension of the membrane has been proposed to be the driver for this, but definitive evidence again remains elusive.^161^ Hence, a fundamental question stays unanswered; what mediates the formation of the β-barrel shape within the membrane?

Release of the nOMP from BAM is preceded by a late-stage intermediate that takes on a B-shape (looking from above/below the membrane) and can be defined as the N- and C-terminal β-strands of the nOMP entering proximity while β-signal engagement is maintained. This allows the nOMP to circularize, separating itself from BAM via a zipper-like mechanism of β-strand complementation between its terminal strands, which outcompetes the BamA β1:OMP β-signal interaction.^142^ But is β-strand complementation enough to facilitate the transition from metastable hybrid barrel intermediate to separated BAM:nOMP structures? EspP has been shown to release from BAM in a more regulated like process, with charged residues and local membrane distortion thought to contribute. With BAM a known membrane disruptor specifically around the β-signal engagement site, is the perturbation of the local membrane a key component in facilitating this transition, or just one of many environmental factors involved? Finally in terms of the OMP folding mechanism, the sequential release of the nOMP must be accompanied by BAM gradually transitioning from a Lateral Open to Lateral Closed state.^142^ How this occurs is also unknown, including whether this involves conformational states of BAM that have not been captured structurally to date.

The relative paucity of studies of BAM activity in physiological contexts in the OM means that much remains unknown about how the membrane and periplasmic environment modulate its function. Recent EPR studies of BAM in bacteria showed that BAM is indeed dynamic in the OM and that Darobactin inhibits these dynamics.^154,176^ How the highly ordered and densely packed membrane affect BAM function is unclear. Several important questions remain unanswered: does BAM fold OMPs into lipid or protein rich domains of the membrane, and how does assembly into BAM ‘precincts’^97^ affect BAM function? Following folding, how is the coordination and organization of OMPs controlled to ensure proper assembly of the OM, including the avoidance of membrane defects upon insertion into the rigid membrane? Are OMP-lipoprotein complexes assembled concurrent with or after OMP folding, and how is such assembly achieved? More broadly, how BAM function is linked to cellular processes such as DNA replication, peptidoglycan remodelling and cell division remain uncertain. Indeed, BAM appears to sit at the hub of many signaling networks, including cell envelope stress responses, but much more research is required to understand these interlinked processes.

Much of the work on the mechanism of BAM has focused on the *E. coli* complex, but given the conservation of BamA across diderms, it remains likely that its general folding mechanism is also conserved. More divergent bacteria have adapted BAM to respond to differences in their cell envelope context and environment, which is reflected in alterations in the lipoprotein constituents of the BAM complex. Despite the diversity in BAM across diderm bacteria, many major pathogens contain BAM complexes that closely resemble that of *E. coli* () and hence these species likely have similar mechanisms of BAM-mediated OMP biogenesis to that described here. What is clear is that we have learned much about the important and fascinating question of how diderms fold OMPs into their OM over the last twenty years that have exploited clever use of biochemical, structural, biophysical analyses *in vitro* and *in vivo* to trap OMPs in the act of folding on BAM. In recent years, molecules targeting BAM have been exploited to study its mechanism of action in OMP folding but these have limited efficacy against major pathogens. Our continually advancing understanding of BAM mechanisms will hopefully open future avenues to develop urgently needed antimicrobials that exploit this structural and mechanistic understanding.

## Supplementary Material









## Notes

S1 – Chemical Structures of Darobactin and Dynobactin. S2 – Alphafold3 predictions of BAM:SurA complexes from all diderm WHO priority pathogen genera. S5 – Supporting Table 3: Uniprot codes for all proteins with structure predictions shown in S2. (PDF)
S3 – Supporting Table 1: PDB and CryoEM information for all structures discussed. (XLSX)
S4 – Supporting Table 2: In vivo phenotypes of BAM subunit deletions. (XLSX)
S6 – Supporting Movie 1: Conformational change of the BAM complex from lateral closed → lateral open → lateral wide-open. (MP4)
