# Supplementary material for: Integration of Membrane Proteins into the Outer Membrane of Diderm Bacteria by the BAM Complex
Source: Chem Rev. 2026 Mar 24;126(7):4036–61. doi: 10.1021/acs.chemrev.5c00764 (PMC13067283; doi:10.1021/acs.chemrev.5c00764)
Supplement: Supplementary file 1 [file cr5c00764_si_001.pdf]

Integration of Membrane Proteins into the Outer Membrane of Diderm Bacteria by the BAM Complex

-

Daniel Birtles[$], Katherine L. Fenn[$], Jonathan M. Machin[$], Sheena E. Radford[*] and Neil A. Ranson[*]
Astbury Centre for Structural Molecular Biology, School of Molecular and Cellular Biology, Faculty of Biological Sciences, University of Leeds, Leeds, LS2 9JT, UK

[$]co-first authors, all contributed equally

*corresponding authors (s.e.radford@leeds.ac.uk; n.a.ranson@leeds.ac.uk)

Supporting Information

S1 – Chemical Structures of Darobactin and Dynobactin
S2 – Alphafold3 predictions of BAM:SurA complexes from all diderm WHO priority pathogen genera
S3 – Supporting Table 1: PDB and CryoEM information for all structures discussed
S4 – Supporting Table 2: In vivo phenotypes of BAM subunit deletions
S5 – Supporting Table 3: Uniprot codes for all proteins with structure predictions shown in S2.
S6 – Supporting Movie 1: Conformational change of the BAM complex from lateral closed → lateral open → lateral wide-open.

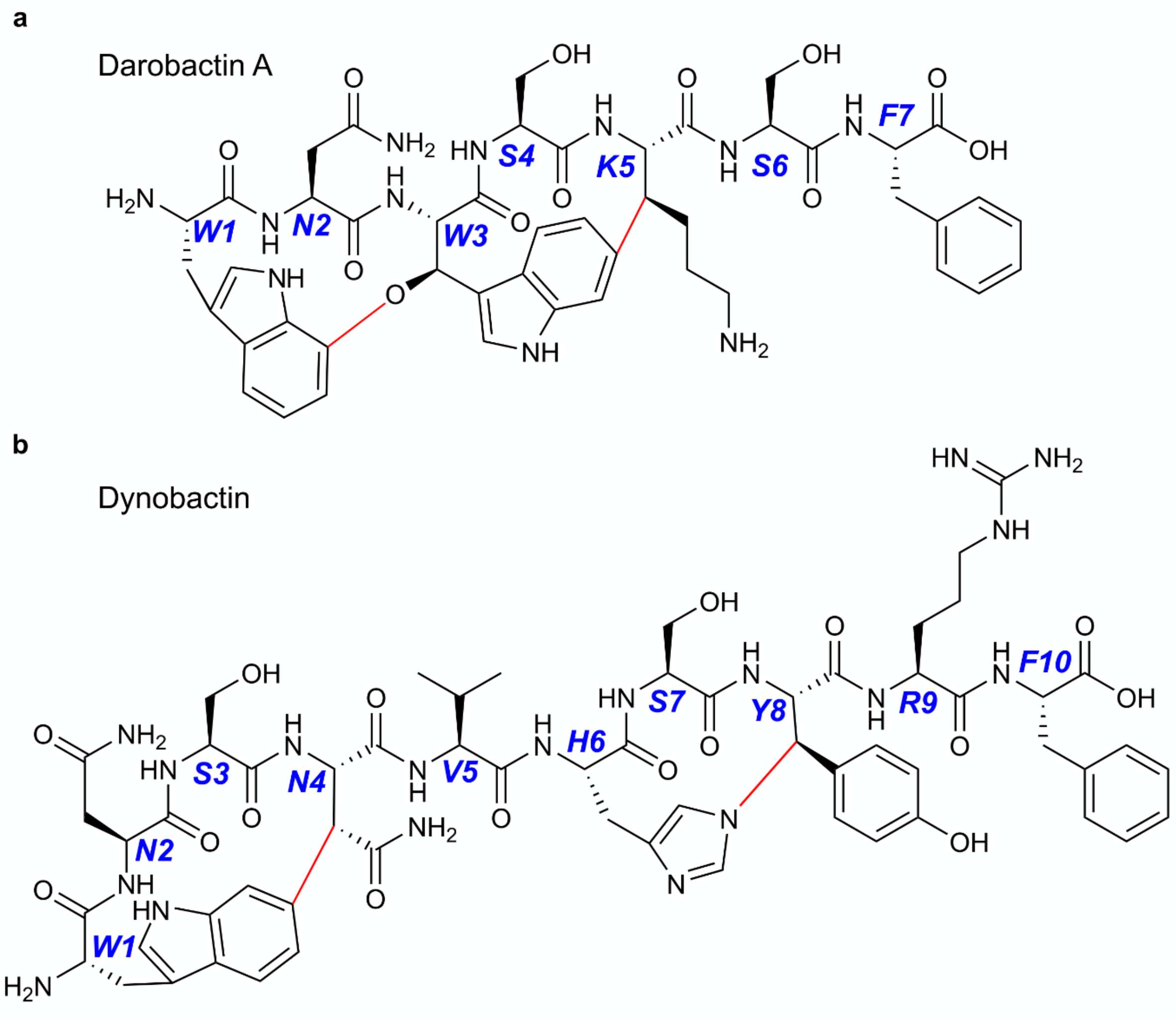

Supporting Information S1: **Chemical structures of (a) Darobactin-A, and (b) Dynobactin.** The peptide sequence and numbers are labelled in blue. The cyclisation crosslinks between amino acid sidechains are shown in red.

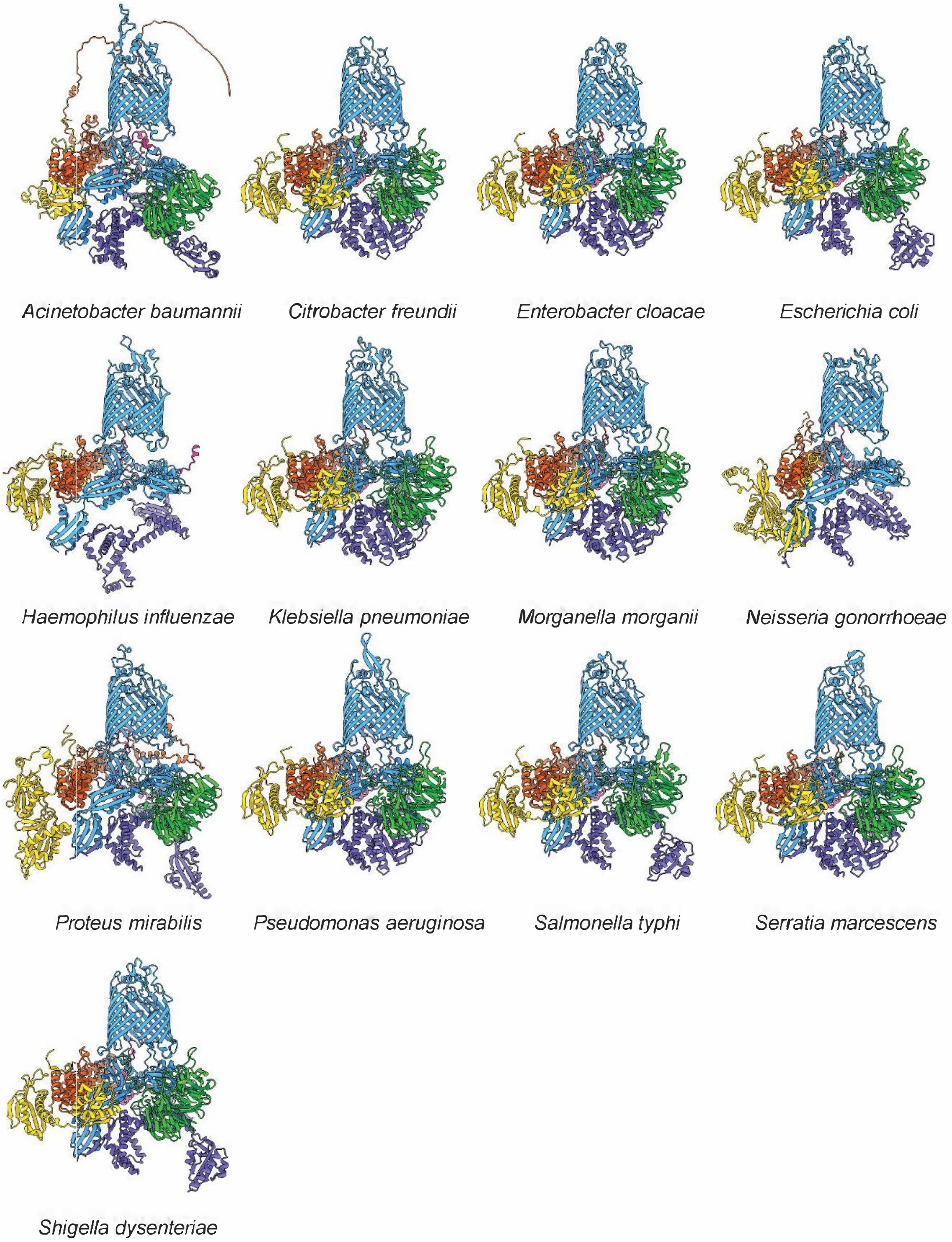

Supporting Information S2: Alphafold3 predictions of BAM:SurA complexes from all diderm WHO priority pathogen genera. The highest priority species was chosen where multiple genera occurred and where a specific species was not indicated the most common species was utilised. Proteins were identified by sequence and structural homology compared to their *E. coli* counterparts. The individual proteins and the complex architecture is structurally well conserved across all species. The BamA of all 13 has 5 POTRAs with a β-augmentation interaction at POTRA-1 with SurA Interactions on POTRA-5 are maintained by BamD and BamE, and POTRA-2 interactions with BamD are also conserved. BamB where present interacts at POTRA-3 but is absent in *H. influenzae* and *N. gonorrhoeae* which also lack PPlase-1 in SurA. Subunits are colored BamA (blue), BamB (green), BamC (yellow), BamD (orange), BamE (magenta) and SurA (purple). (The list of each protein used is in S5 - Supporting Table 3.

S3 – Supporting Table 1: PDB and CryoEM information for all structures discussed
HYPERLINK TO CONTENT OF:
"S3 Supporting Table 1 -structural data summary.xlsx"

S4 – Supporting Table 2: In vivo phenotypes of BAM subunit deletions
HYPERLINK TO CONTENT OF:
"S4 Supporting Table 2 - Invivo_deltabamBCDE_Phenotypes.xlsx"

Species	BamA	BamB	BamC	BamD	BamE	SurA
Acinetobacter baumannii	A0A7S8WB78	A0A6F8TKL1	A0AA45B997	A0A1E3M1K3	A0A0M3FHZ0	A0A429LR97
Citrobacter freundii	A0A859T7H0	A0A7G2IZA3	A0A859TJK4	A0A7D6Z648	A0A7G2IYJ2	A0A243U1T9
Enterobacter cloacae	A0A6S5JMV2	A0A6S5JQM1	A0A6S5K0U2	A0A7H8UEA5	A0A0X4AWL0	A0A6S5JFQ9
Escherichia coli	P0A940	P77774	P0A903	P0AC02	P0A937	P0ABZ6
Haemophilus influenzae	P46024	-	A0AAE8D2C4	A0A2S9RQB9	A0A2S9S3M2	A0A2S9RCD0
Klebsiella pneumoniae	A0A8D6Q4R0	A0A486R062	A0A7X1HU83	A0A9Q4RR01	A0A5D3JF05	A0A9J6RV86
Morganella morganii	J7TNX3	J7U6S6	J7TFW8	M1SDT4	M1SEW5	J7TNT2
Neisseria gonorrhoeae	P95359	-	A0A1D3J262	Q50985	A0A1D3EW25	A0A5K1Q9W5
Pseudomonas aeruginosa	A0A232C799	A0A3S0IXX2	A0A3S0LDF5	A0A509JJN6	A0A9Q9K074	A0A5E5R595
Proteus mirabilis	A0A1Z1SSX0	A0A1Z1STA0	A0A1Z1SUX5	A0A1Z1SXR8	A0A1Z1ST44	A0A1Z1T0D1
Salmonella typhi	Q8Z9A3	A0A716WIB5	A0A748ESA5	A0A3Z6T0V7	A0A751N435	Q8XEV3
Serratia marcescens	A0A2S4X5B9	A0A2V4FU69	A0A2S4X671	A0A086FBJ7	A0A084X0E5	A0A2V4FZB9
Shigella dysenteriae	A0A3R0X394	A0A403M3S6	A0A403M4Y4	A0A403M6N3	A0A2S8E8A5	A0A1Q8NQ11

Supporting Information S5 – Supporting Table 3: Uniprot codes for all proteins with structure predictions shown in S2.

S6 – Supporting Movie 1: Conformational change of the BAM complex from lateral closed → lateral open → lateral wide-open.
PDBs used in creating the movie: lateral closed - 5D0O, lateral open - 9CNW, lateral wide-open - 8QPV
HYPERLINK TO CONTENT OF:
"S6_Supporting Movie 1.mp4"
